# Studying the influence of single social interactions on approach and avoidance behavior: A multimodal investigation in immersive virtual reality

**DOI:** 10.3758/s13428-025-02627-0

**Published:** 2025-04-25

**Authors:** Sabrina Gado, Matthias Gamer

**Affiliations:** Department of Psychology, Experimental Clinical Psychology, Julius-Maximilians-Universität Würzburg, Marcusstr. 9-11, 97070 Würzburg, Germany

**Keywords:** Social attention, Social conditioning, Vigilance-avoidance, Virtual reality, Ecological validity

## Abstract

When studying spontaneous or learned emotional responses to social stimuli, research has traditionally relied on simplified stimuli repeatedly presented on a computer screen in standardized laboratory environments. While these studies have provided important insights into social perception and cognition, their restricted ecological validity may impede the extrapolation of findings to everyday social contexts. Here, we developed a novel immersive virtual reality scenario that permits the examination of social approach and avoidance behavior under naturalistic circumstances while at the same time maintaining full experimental control. Using a combination of a social conditioning procedure with a social approach–avoidance test, we conducted two experiments (both with *N* = 48 female participants) to investigate how individuals differing in trait social anxiety adapt their behavior after a single encounter with an either friendly or unfriendly virtual agent. In addition to overt approach and avoidance behavior, we acquired subjective ratings, eye-tracking data, and autonomic responses. Overall, we observed significant effects of the social conditioning procedure on autonomic responses and participants’ exploration behavior. After initially increased attention, participants exhibited avoidance of social threats as indicated by a higher interpersonal distance and decreased visual attention towards the negatively conditioned virtual agent in the test phase. We found no association between hypervigilance and trait social anxiety but observed higher fear ratings and enhanced avoidance of social threats in participants with elevated anxiety levels. Altogether, this study demonstrates the potential of immersive virtual environments for examining social learning processes under conditions resembling real-life social encounters.

**Supplementary Information:**

The online version contains supplementary material available at 10.3758/s13428-025-02627-0.

## Introduction

For most mammals, daily life requires the adaptive regulation of emotions and behaviors in social situations (Bechara et al., 2000; Frijda, 1986; Griffiths & Scarantino, 2012). Since humans are a highly social species, only adherence to the prevailing norms and rules of society allows us to be part of a community (Young, 2015). Individuals who do not behave in a socially appropriate manner are at risk of exclusion which—at least in the past—could lead to reduced access to critical resources like food, protection, and mating opportunities, ultimately posing a significant risk to survival. Thus, adaptive social approach and avoidance behavior is of utmost importance for social functioning (Cacioppo & Hawkley, 2009; Eisenberger & Cole, 2012; Holt-Lunstad et al., 2010). From early childhood and continuing into adulthood, humans must therefore be able to quickly learn and adopt behaviors, attitudes, and beliefs from the social environment in which they live (Fonagy et al., 2022; Reeb-Sutherland et al., 2012; Sheridan et al., 2018). The presence of or interaction with other human beings is a highly potent stimulus for triggering approach or avoidance tendencies that can be flexibly shaped by learning experiences. Understanding the consequences of social encounters is hence an important mechanism for shaping one’s behavior regarding approach and avoidance tendencies for interaction partners associated with reward or threat, respectively (Davis et al., 2010; Lin et al., 2012; Lissek et al., 2008; Wieser et al., 2014).

Although these learning processes are usually adaptive, allowing a person to flexibly adjust to current situational demands based on previous experiences, they can also go awry (Pejic et al., 2013). Indeed, an imbalance in social approach and avoidance tendencies may constitute a risk factor for the etiology and maintenance of mental disorders characterized by deficits in social functioning (Struijs et al., 2018), such as social anxiety disorders (SAD). SAD is characterized by a persistent fear of negative evaluation and humiliation in social situations, such as conversations, performing in front of others, and being observed (American Psychiatric Association, 2013; Clark & Wells, 1995; Stein & Stein, 2008). The lifetime prevalence of SAD was estimated to be 6.7% among European populations (Fehm et al., 2005) and about 10.7% in the United States (Kessler et al., 2012). In a recent international study, around one-third of individuals aged 16 to 29 self-identified as meeting the criteria for SAD (Jefferies & Ungar, 2020). Overall, women are more affected than men (Asher et al., 2017; McLean et al., 2011). Given the substantial individual and societal burden, it is essential to elucidate the mechanisms underlying the etiology of SAD and other disorders that significantly impair social functioning. Such research is also crucial for improving current psychotherapeutic interventions that—although generally effective—still demonstrate reduced effects or high relapse rates in some patients (Leichsenring et al., 2014; Mayo-Wilson et al., 2014; van Dis et al., 2020).

A common assumption is that prior aberrant learning experiences play a key role in the etiology of anxiety disorders as well as in processes contributing to their maintenance (LeDoux & Pine, 2016; Lonsdorf et al., 2017; Mineka & Oehlberg, 2008). Individuals with SAD have likely experienced negative or traumatic social events, such as criticism, rejection, or humiliation. These encounters can contribute to a pervasive fear of social situations, resulting in heightened sensitivity to social cues and a tendency to avoid social interactions. Indeed, avoidance might be an effective short-term strategy to regulate the fear response and is therefore potentially reinforced as a useful coping mechanism. However, this tactic most likely causes anxiety to persist or increase in the long run, as there is no exposure to situations with a positive outcome to correct negative expectations (Pittig et al., 2023). To investigate the behavioral, attentional, and physiological mechanisms involved in the development and persistence of anxiety disorders, conditioning procedures serve as a valuable research paradigm (Haaker et al., 2019; Lonsdorf et al., 2017).

In this context, Toth and colleagues (2012) developed a social fear conditioning procedure for rodents to investigate neurobiological mechanisms related to social fear acquisition and extinction in mice. This paradigm was adopted for humans by Shiban et al. (2015). The authors used virtual reality (VR) to overcome the problem of low ecological validity in traditional laboratory paradigms, where social situations are often modeled by presenting static pictures of faces (e.g., Lissek et al., 2008; Wieser et al., 2014) or short video clips (e.g., Wiggert et al., 2017). At the same time, VR offers a higher degree of experimental control and between-subject standardization as compared to experiments including real persons, such as confederates (Bell et al., 2020). These field-like experiments are associated with high costs, logistical efforts, and reduced control and consistency (Blascovich et al., 2002). Therefore, VR is a promising avenue for investigating social conditioning in humans, as it offers a unique opportunity to simulate complex social situations and, at the same time, provide a high level of experimental control (Andreatta et al., 2023; Parsons, 2015).

By selectively manipulating key components within virtual social situations, researchers can investigate how individuals learn to associate social stimuli with threat or reward, and how they adapt their own behavior. This applies to components like the (social) context (Glotzbach et al., 2012; Marusak et al., 2017), the behavior of virtual interaction partners comprising verbal utterances (Shiban et al., 2015), facial expressions (Lange & Pauli, 2019), direction of gaze (Kyrlitsias et al., 2020; Rubo & Gamer, 2021), and gestures (Bailenson & Yee, 2005; Pavlidou et al., 2023), as well as the role, position, and appearance of the participants within the virtual environment (Banakou et al., 2013, 2018; Yaremych & Persky, 2019; Yee & Bailenson, 2007). Furthermore, previous studies have provided first evidence that trait social anxiety modulates these learning processes and affects people’s social approach and avoidance behavior in VR concerning place preferences (Kiser et al., 2022), interpersonal distance (Lange & Pauli, 2019), and visual attention (Reichenberger et al., 2020). In general, these findings are mostly consistent with the so-called vigilance–avoidance hypothesis (Chen & Clarke, 2017). According to this theory, individuals with SAD initially show heightened attention towards external indicators of negative social evaluation (Heimberg et al., 2014), with subsequent strategic avoidance of social cues to reduce emotional distress (Salkovskis, 1991). Although prior research has provided important insights into social learning processes and their influence on behavior, it still exhibited notable limitations in terms of ecological validity. This also applies to previous experiments in VR concerning the following shortcomings: First, most previous studies utilized relatively bland virtual environments that differ substantially from naturalistic situations, which poses a significant problem for the interpretation of some outcome measures. For example, attentional bias towards threatening or emotional stimuli is contingent on the presence of competing targets (Wirth & Wentura, 2018), and attentional exploration in general is strongly affected by the visual complexity of the scenario (Wu et al., 2014). Simplistic environments may fail to provide such competition, thereby constraining the validity of conclusions drawn about alterations of attentional dynamics. By contrast, naturalistic and enriched environments are considered more suitable for eliciting authentic behavior, thus enabling more meaningful insights into real-world actions, which inherently unfold in complex and dynamic contexts (Tatler et al., 2011). Furthermore, participants’ presence in the environment is an important factor that fosters more natural behavior in the VR—and more complex environments elicit higher presence (Parsons et al., 2017). In the present study, we thus employed a realistic and visually enriched environment to strike a balance between ecological validity and experimental control, enabling the robust observation of attentional biases while carefully accounting for potential confounding effects. Second, previous experiments mostly employed conventional conditioning procedures that comprised multiple acquisition trials. Such procedures do not necessarily resemble real-life social scenarios where already one brief interaction might substantially affect impression formation (Ambady & Rosenthal, 1992) and thereby potentially also impact one’s behavior. Third, participants’ behavior in previous studies was often restricted to unnatural movement, for example, using joystick control. Behavioral effects may therefore not be representative of natural movements in everyday social situations.

Here, we present a novel virtual environment combining a highly naturalistic social conditioning procedure (Toth et al., 2012) with a social approach–avoidance test (Landauer & Balster, 1982). In this environment, we conducted two experiments to investigate whether participants adopt flexible approach and avoidance tendencies towards differently conditioned virtual agents, being computer-controlled virtual characters, on a behavioral level (i.e., whole-body movement, interpersonal distance), regarding active exploration (i.e., gaze behavior) as well as on a subjective level (i.e., subjectively perceived likeability, fear, and anger). We further examined differences in autonomic responding (pupillary, electrodermal, and cardiovascular responses). In the first experiment, the social approach–avoidance test was implemented with stationary conditioned virtual agents, whereas in the second experiment the conditioned virtual agents were moving. This forced the participants to adapt their behavior more dynamically.

The previously discussed *vigilance*–*avoidance* model (Chen & Clarke, 2017; Reichenberger et al., 2020) proposes two behavioral strategies which people—especially those with high social anxiety—use to cope with potentially threatening social stimuli: (Hyper-)vigilance refers to a state of enhanced alertness and sensitivity to potential threats or dangers in the environment (Richards et al., 2014). It encompasses, for instance, so-called hyperscanning, the excessive and intense monitoring of social cues, such as others’ facial expressions and body language, as indicators of evaluation or criticism (Horley et al., 2003). Vigilance allows people to quickly identify potential negative judgment and to adjust their behavior accordingly. Avoidance, on the other hand, helps people to reduce emotional distress induced by socially threatening stimuli (Hofmann & Hay, 2018; Mowrer, 1939; Salters-Pedneault et al., 2004). This would be reflected in a larger interpersonal distance, a reduced amount of time spent in close proximity to a virtual agent, and reduced visual attention towards the body and face of a negatively conditioned versus positively conditioned or neutral virtual agent. In contrast, symptoms of (hyper-)vigilance would be heightened visual attention towards the virtual agents’ body and face. We expected that trait social anxiety further amplifies these effects, such that participants with high social anxiety show even more avoidant/hypervigilant behavior towards a negatively conditioned versus the positively conditioned and neutral virtual agents. Besides the behavioral strategies, we were also interested in whether participants learn to differentiate between the virtual agents on a subjective experience level as operationalized by explicit ratings of likeability, fear, and anger. Finally, we explored differences in autonomic responding. We expected that negative social experiences would directly elicit brief reductions in heart rate (bradycardia) along with increased skin conductance and pupil diameter—body reactions characterizing threat-related defensive states (Lonsdorf et al., 2017). In the following approach–avoidance test, participants were supposed to exhibit higher autonomic arousal when in close proximity to a virtual agent who previously showed unfriendly behavior, as opposed to when being in close proximity to a virtual agent who behaved kindly. Both effects were expected to be even more pronounced in participants with high social anxiety. Further details can be found in the preregistration of the current study (https://osf.io/7cxdh).

## Methods

We report how we determined our sample size, all data exclusions, all experimental manipulations, and all measures in the study, and the study follows the Journal Article Reporting Standards (JARS). A demonstration video, supplementary material, aggregated data, and the code for all analyses and plots are openly available on the Open Science Framework (OSF) at https://osf.io/mc85y/. The two VR games and raw data are available from the lead contact upon request.

### Participants

Sample size planning was done prior to data collection. To achieve a complete between-subject randomization, assigning four distinct roles (friendly, unfriendly, neutral, and an unknown control condition) to four virtual agents, and allocating the rooms in the test phase (dining and living room), a minimum sample size of 4! × 2 = 48 was required. Several power analyses were performed for paired-samples *t*-tests (friendly vs. unfriendly condition) with a significance level of α = .05, using the descriptive statistics of a pilot study to explore the achievable statistical power for detecting meaningful effects in the interpersonal distance (one-sided *t*-test), the time spent in close proximity to the conditioned virtual agents, and the head fixations (both two-sided *t*-tests) with a sample size of 48. Based on these analyses, a sample size of 48 was considered adequate to detect the specified effects. Notably, this sample size is comparable to previous similar VR studies (Reichenberger et al., 2017, *N* = 43, Reichenberger et al., 2020, *N* = 53; Welsch et al., 2018, *N* = 40, Welsch et al., 2020, *N* = 76). For both experiments, participants were recruited until we had 48 valid data sets, accounting for potential exclusions due to recording issues, motion sickness, or impaired memory regarding the roles of the virtual agents (see below). The samples of both experiments were independent. Data were collected in July and August 2023 (Experiment 1), and from October 2023 until January 2024 (Experiment 2).

Participants were recruited primarily through the university’s participant management system, meeting specific inclusion criteria. Eligible participants were required to be female, be aged between 18 and 40 years, be proficient in both spoken and written German, and possess normal or corrected-to-normal vision using contact lenses or (small) glasses. The restriction on eyewear size was necessary, as the head-mounted display (HMD) used for presenting the virtual environment was incompatible with larger glasses. To ensure a wide range of social anxiety traits, we also extended invitations to individuals who had previously undergone prescreening for SAD severity as part of a different study and had consented to further contact based on their questionnaire responses (Gado et al., 2024). To reward them for their effort and time, participants received either monetary compensation or course credit. The study was conducted according to the Declaration of Helsinki and was approved by the ethics committee of the Department of Psychology of the University of Würzburg (GZEK-2023-17).

To obtain 48 valid observations, we had to recruit 64 participants for the first experiment. The exclusions were due to recording problems and technical issues (*n* = 5) or considerable symptoms of motion sickness exceeding the sample mean of the simulator sickness questionnaire by more than one standard deviation (*n* = 9). Additionally, we excluded participants who forgot or confused the virtual agents’ previous behavior (*n* = 2). Therefore, we asked them to assess the perceived friendliness of the agents’ previous behavior on a scale of 0 to 100. Only participants who rated the friendly agent at least 10 points higher than the unfriendly one were included in the study. The included participants were all female and had a mean age of 23.1 years (*SD* = 3.3, range = 18–32). We chose to recruit only female participants and used only male virtual agents, as we anticipated this particular setup to predominantly evoke fear, rather than anger (Reichenberger et al., 2019; Wieser et al., 2010).

In the second experiment, we recruited 70 participants, of which 22 participants had to be excluded (*n* = 7 due to recording problems and technical issues, *n* = 6 because of symptoms of motion sickness exceeding the sample mean of the simulator sickness questionnaire by more than one standard deviation, and *n* = 9 who forgot or confused the virtual agents’ previous behavior). The final sample of 48 participants (all female) had a mean age of 23.5 years (*SD* = 4.0, range = 19–37).

Although it is often difficult to obtain high levels of anxiety traits in community samples, we achieved a satisfactory range of social anxiety in both experiments (see Fig. S1). Experimenters were blind to the prescreening social anxiety scores of participants.

### Apparatus

During the experiments, participants were fully immersed in the virtual environment. The VR application was implemented in Unreal Engine 5.1.1 using the MetaHuman framework to create and animate the virtual agents (Epic Games, Cary, NC, USA). A demonstration video illustrating the virtual environment and the experimental procedure is available in our OSF repository (https://osf.io/nx4qv).

The environment was displayed on an HP Reverb G2 Omnicept (Hewlett-Packard, Palo Alto, CA, USA) head-mounted display (HMD) with a resolution of 2160 × 2160 pixels per eye, a refresh rate of 90 Hz, and a 114° field of view. The built-in eye tracker (Tobii AB, Danderyd, Sweden) recorded participants’ gaze direction and pupil diameter from both eyes at a sampling rate of 120 Hz and accuracy of < 1° visual angle. Sounds were presented over off-ear headphones attached to the HMD (Valve, Bellevue, WA, USA). The experiment ran on a Windows 10 64-bit machine with an Intel Core i9-9900K, 32 GB RAM, and an Nvidia GeForce RTX 2080 Ti. To enable natural 360-degree walking throughout the virtual environment, we used a Cyberith Virtualizer ELITE 2 omnidirectional treadmill (Cyberith Virtualizer, Vienna, Austria).

Physiological signals (electrodermal activity and electrocardiogram) were recorded with mobile electrocardiography (ECG) and electrodermal activity (EDA) sensors (Movisens, Karlsruhe, Germany). Electrocardiographic activity data were collected at a sampling rate of 1024 Hz using the Movisens EcgMove 4 sensor, which was positioned on the left side of the sternum, just below the left breast of the participants. Electrodermal activity was recorded with the Movisens EdaMove 4 sensor at a sampling rate of 32 Hz. We followed the guidelines by Nebe et al. (2023), and participants’ skin was prepared using lukewarm water (no soap, alcohol, or abrasion) and the electrodes were attached to the thenar and hypothenar eminences of the participants’ non-dominant hand.

### Procedure

**Fig. 1:**
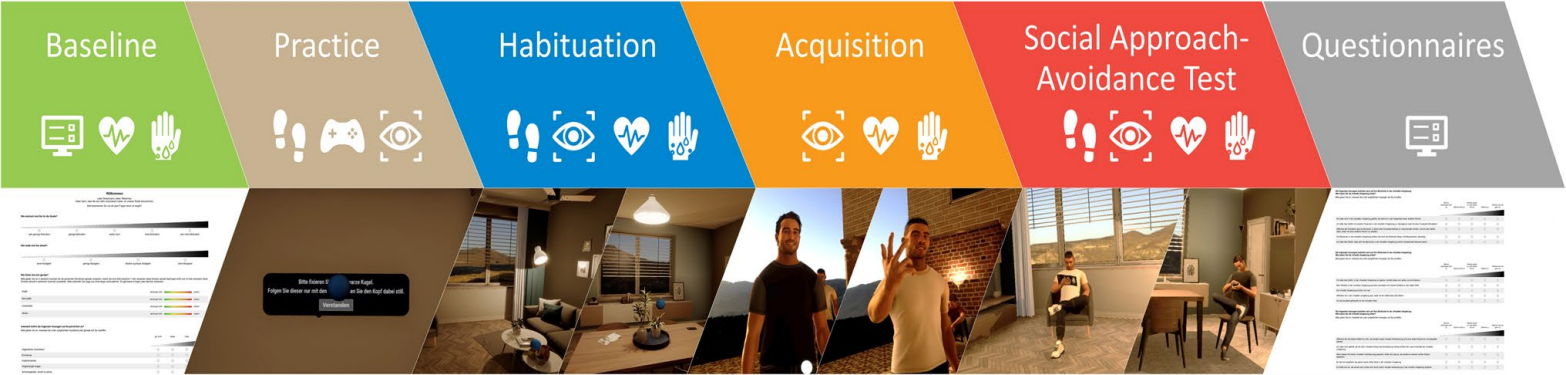
Schematic representation of the experimental procedure. First, we acquired resting-state data for heart rate and electrodermal activity, as well as ratings of tiredness, motivation, state anxiety, nervousness, distress, and stress. During the practice phase, participants learned how to walk on the omnidirectional treadmill and how to use the controller. During the habituation phase, participants undertook a guided room tour to become acquainted with the environment, followed by the opportunity to freely explore and later rate the virtual rooms. In the acquisition/social conditioning phase, participants encountered three virtual humans on the terrace of the flat. Approaching them elicited a positive reaction (smile, positive utterance), a negative reaction (aggressive facial expression, negative utterance), or a neutral reaction (short look). During the social approach–avoidance test, participants could again freely explore the rooms. In Experiment 1, the previously negatively conditioned virtual agent was sitting in one of the rooms while the previously positively conditioned virtual agent was sitting in the other room, whereas in Experiment 2, the virtual agents were walking around freely during the test phase. Afterwards, the participants were asked to rate the rooms, and then rate the different virtual agents regarding likeability, fear, anger, attractiveness, and how they perceived their previous behavior. At the end of the experiment, participants completed questionnaires on their trait social anxiety (SPAI & SIAS), anxiety sensitivity (ASI-3), social skills (ISK), autism spectrum traits (AQ-K), and state anxiety, nervousness, distress, and stress. We also acquired data on their experienced motion sickness (SSQ) and presence (IPQ & MPS) in the virtual environment

The experiment consisted of six phases (see Fig. 1 for an overview). After their arrival in the laboratory, participants read and signed the informed consent and filled out a short questionnaire on their current tiredness, motivation, and emotional state (anxiety, nervousness, distress, and stress; visual analogue scales ranging from 1 to 10). After equipping participants with the mobile ECG and EDA sensors, we conducted a one-minute resting-state measurement, where participants were instructed to sit calmly. Afterwards, participants were placed in the center of the omnidirectional treadmill and taught how to walk on the platform (see Fig. S2). They were then asked to put on the HMD, and the proprietary HP nine-point eye-tracking calibration routine was carried out. The actual experiment commenced in a separate virtual room, providing the opportunity for participants to acquaint themselves with the virtual environment, practice navigation, and become familiar with the controller to answer questions and confirm instructions throughout the experiment (*practice phase*). Furthermore, an additional five-point eye-tracking validation was performed within the virtual environment. If this validation had failed for any participant, we would have repeated the eye-tracking calibration (see Fig. S3 for the validation results of both experiments).

For the start of the experiment, participants were teleported to the virtual flat constituting the experimental environment to complete the *habituation phase*. They were told to imagine being invited to their friend’s vacation home, but unfortunately, their friend was currently occupied with grocery shopping. Nevertheless, the participants would be given a tour of the virtual environment in their friend’s absence. At this point, a small white arrow popped up in the virtual environment, serving as their interactive guide. As participants approached the arrow, it continued its prespecified path directing them through the entire flat consisting of three rooms (office, living room, and dining room; see Fig. S4 for a floor plan). We randomized whether the arrow started towards the living room or the dining room. Upon completion of the room tour, participants should have acquired an initial understanding of the virtual environment. Subsequently, they were given an additional three minutes to freely navigate and explore the virtual flat. After this, participants were teleported to each room and asked to indicate how comfortable they were currently feeling in this room.

In the following *acquisition/social conditioning phase*, they were informed about other guests waiting on the terrace and were invited to say “Hello” to them. Following their confirmation, they were teleported to the terrace, where they encountered three virtual agents. Participants were instructed to initiate interactions by clicking on the virtual agents and to pay attention to their reactions. The clicks elicited the following reactions: The friendly virtual agent approached the participants with a warm greeting, saying, “Hello, I am Max. Nice to meet you!” before going inside. The unfriendly virtual agent exhibited an antagonistic response. Approaching the participants, he angrily exclaimed, “What the heck are you looking at? Get lost!” before also entering the flat. Meanwhile, the neutral virtual agent remained seated and was busy with his smartphone. Upon interaction initiation, he briefly acknowledged the participant’s presence with a glance before resuming his smartphone activities.

Subsequently, participants were teleported back inside the flat, and the door leading to the terrace was closed behind them. After observing the friendly and the unfriendly virtual agent each entering a different room (living room and dining room), they were informed about now having an additional three minutes to freely explore the flat (*social approach–avoidance test*). In Experiment 1, the two virtual agents remained seated in the rooms: one busy using a smartphone in the dining room, and the other reading a book in the living room. When the participants entered a room, the virtual agents briefly looked at them before resuming their activities. Upon clicking on the virtual agents once more, they would again look towards the participants and respond in accordance with their assigned role, offering either a friendly smile or an unfriendly frown. In Experiment 2, after going into the different rooms, the two virtual agents freely moved around, but they did not respond to the participant’s actions or presence.

When the three minutes had expired, participants were again teleported to each room and asked to indicate how comfortable they were currently feeling in this room. Following this, they were brought back to the starting room, where they were asked to subsequently rate the different virtual agents regarding likeability, fear, anger, attractiveness, and their previous behavior. All ratings were collected for the friendly, unfriendly and neutral agents, and we additionally acquired likeability, fear, anger, and attractiveness ratings for an unknown control agent that had not been met before. This was done to explore unspecific effects of the social conditioning procedure on social evaluation. Lastly, we repeated the five-point eye-tracking validation (see Fig. S3 for the results in both experiments).

After taking off the HMD and leaving the omnidirectional treadmill, participants completed questionnaires inquiring again about their current emotional state as well as sociodemographic information, trait social anxiety, anxiety sensitivity, autistic traits, social skills, and the assumed purpose of the study.

### Measures and data processing

All data processing steps were performed with Python (version 3.10; Van Rossum & Drake, 2009).

#### Questionnaires

Our primary measure of social anxiety in this study was the German version of the Social Phobia and Anxiety Inventory (SPAI; Fydrich, 2016). We further assessed symptoms of motion sickness using the Simulator Sickness Questionnaire (SSQ; Kennedy et al., 1993). Participants who reported a substantial increase in motion sickness symptoms from before to after the experiment were excluded from the analyses. Moreover, the experienced presence was captured using the Igroup Presence Questionnaire (IPQ; Schubert et al., 2001) and the Multimodal Presence Scale (MPS; Makransky et al., 2017; Volkmann et al., 2018). For more details on the questionnaires, see the Supplementary Methods (https://osf.io/jmhfa).

#### Ratings

Within the virtual environment, we asked participants three times to rate how comfortable they were feeling in general: First, during the practice phase; second, after the habituation phase; and third, after the social approach–avoidance test. They answered this question on a visual analog scale presented in front of them ranging from 0 (“very uncomfortable”) to 100 (“very comfortable”). After every exploration phase, we also asked participants to rate how they felt inside the different rooms using the same scale.

At the end of the virtual reality experience, participants were asked to rate the four different virtual agents (i.e., friendly, unfriendly, neutral, and unknown agent) regarding likeability (“How likeable is this person to you?” from 0 = “very unlikeable” to 100 = “very likeable”), fear (“How afraid are you of this person?” from 0 = “not at all” to 100 = “very much”), anger (“How much anger do you feel towards this person?” from 0 = “none” to 100 = “very angry”), and attractiveness (“How do you rate the attractiveness of this person?” from 0 = “not attractive at all” to 100 = “very attractive”). Furthermore, for all agents that appeared during the social conditioning phase (i.e., friendly, unfriendly and neutral agent), participants were asked to rate their previous behavior (“How would you characterize this person’s prior behavior towards you?” from 0 = “very unfriendly” to 100 = “very friendly”).

#### Behavior

Apart from self-report measures, we also captured physical behavior, more precisely whole-body movements, in the virtual world. To this aim, the participants’ and the virtual agents’ current positions were recorded at runtime with a sampling rate of about 10 Hz. To correct for varying frame rates, we downsampled the data to 5 Hz. Based on the resulting movement trajectories, we calculated the Euclidean distance between the position of the participant and the positions of the virtual agents for every time point during the test phase. The stationary setting in Experiment 1 allowed us to investigate adaptations in the chosen distances as a result of the social conditioning procedure. Here, we calculated the difference between participants’ chosen distances to the fixed positions of the agents during the habituation and the test phase. To account for the fact that due to the dynamic setting in Experiment 2, participants might not always have been aware of the agents’ proximity, we additionally calculated the interpersonal distance only for phases where the virtual agents were visible to the participants.

We also assessed the duration the participants spent in the same room with the different virtual agents and how often they tried to interact with the virtual agents by clicking on them. In Experiment 1, we could again investigate changes in place preference by calculating the difference between the time participants spent in a room during the habituation and the time they spent there during the test phase. For the analysis of the time participants spent in the different rooms, three participants of Experiment 1 had to be excluded because of technical problems with the necessary event readouts.

#### Eye tracking

To record gaze behavior, we used the integrated eye tracker in the HMD which estimates a gaze vector in real time, and by means of raytracing, allows for identifying which object in the virtual environment is hit by this gaze vector. For each frame, we recorded the name of the attended object. To correct for varying frame rates, we resampled the data to 50 Hz. To acquire a measure of social attention, we then calculated the proportional dwell time on social regions of interest (i.e., the body and face of the virtual agents) for the acquisition and the test phase. As a measure of hyperscanning, we counted how often participants redirected their attention towards a social stimulus (body or face of virtual agent).

#### Physiology

The ECG data were exported from the mobile ECG sensor and then filtered using a 5 Hz high-pass filter. We then used a semiautomatic method as implemented in the *NeuroKit2* Python package (Makowski et al., 2021) to identify R-peaks. The annotated ECG was then visually inspected for plausibility and corrected if necessary; artifacts and noisy sections were removed. In Experiment 1, six participants had to be excluded because they had less than 75% usable data, while the remaining participants had on average 98.84% (*SD* = 3.64%) usable data. We also had to exclude six participants in the second experiment because they also had less than 75% usable data. The remaining participants had on average 99.81% (*SD* = 0.92%) usable data. The average heart rate (in beats per minute; bpm) was calculated using the remaining data based on the R-R intervals.

To extract skin conductance level and responses, we first removed noisy passages using a fast Fourier transform (FFT) approach with sliding windows of varying sizes to automatically identify and remove data spans with artifacts. Next, sections without any signal were also removed from the data. The cleaned signal was then filtered using a 2 Hz low-pass filter, and based on this, we computed the average skin conductance level for different experimental phases. Skin conductance responses were identified as the local minima and maxima with a minimum amplitude of 0.02 µS and a rise time between 0.1 and 10 seconds. In Experiment 1, seven participants had to be excluded because of less than 75% usable data, while the remaining participants had on average 98.08% usable data (*SD* = 3.42%). Again, we also had to exclude seven participants in the second experiment because of less than 75% usable data, and the remaining participants had on average 98.74% usable data (*SD* = 3.75%).

For pupillary responses, the raw pupil diameter was downsampled to 50 Hz. Phases with more than 25% missing data were excluded. No participants had to be excluded because of more than 25% missing data for the whole experiment. In Experiment 1, participants had an average of 89.81% usable data (*SD* = 3.01%); in Experiment 2, the proportion of usable data was 88.23% (*SD* = 3.09%). Data were then linearly interpolated for data spans with missing data or blinks and filtered using a low-pass filter with a cutoff frequency of 5 Hz and a roll-off of 12 dB/octave.

All physiological measures were baseline-corrected using the average values of the last 30 seconds of the practice phase.

### Statistical analyses

All statistical analyses were performed in Python (version 3.10; Van Rossum & Drake, 2009). Linear mixed models (LMMs) were implemented using the *Pymer4* package (version 0.8.1; Jolly, 2018) and mainly included the fixed effects “behavior of the virtual agent” and “level of social anxiety” (as measured with the SPAI questionnaire). Moreover, all analyses included a random intercept for participants to account for the manipulation of conditions within subjects. To quantify the main and interaction effects of the LMMs’ fixed effects, we report *F*-tests with type III sum of squares, orthogonal polynomial contrasts, and Satterthwaite approximated degrees of freedom for all models.

Concerning the rating data (i.e., likeability, anger, fear), we were interested in the main effects of the “behavior of the virtual agent” (friendly vs. unfriendly vs. neutral vs. unknown) and the “level of social anxiety” as well as their interaction effect.

To explore the effects on physiological responses, we examined the changes in heart rate, pupil diameter, and skin conductance responses during the acquisition phase. Significant effects of the experimental conditions and trait social anxiety in the temporal patterns were identified using cluster-based permutation tests (Ehinger, 2016; Maris & Oostenveld, 2007). Therefore, we resampled heart rate, pupil diameter, and skin conductance at a frequency of 10 Hz and computed repeated-measures analyses of covariance (ANCOVAs) with the factors “behavior of the virtual agent” (friendly vs. unfriendly) and “level of social anxiety” at every time point. Based on these individual tests, we were able to identify contiguous clusters. Subsequently, we evaluated the likelihood of such clusters by using our data but shuffling the condition labels within participants (random partitioning) and then repeating the identification of contiguous clusters (1000 permutations). If the probability of a cluster resembling ours, or an even more extreme, occurring in the absence of any actual difference between conditions was less than 5%, the identified cluster was deemed significant.

For the acquisition phase, we additionally analyzed participants’ visual attention. Therefore, we calculated the proportional dwell time on the body and the face of the virtual agents, and the extent of hyperscanning operationalized as shifts in visual attention towards a social stimulus (body or face of virtual agent). For both measures, we computed LMMs with the fixed effects “behavior of the virtual agent” (friendly vs. unfriendly) and “level of social anxiety.”

For the subsequent test phase, we performed similar LMMs with the fixed effects “behavior of the virtual agent” (friendly vs. unfriendly) and “level of social anxiety” and the dependent variables proportional dwell time and extent of hyperscanning. Please note that our design only allowed for a comparison of the friendly and unfriendly agent with regard to physiological responses, gaze, and behavior in the acquisition and test phase because only those agents showed comparable behavior except for the “friendliness.”

Behavioral adaptations following the social conditioning procedure were examined by utilizing LMMs with the fixed effects “phase” (habituation vs. social approach–avoidance test), “behavior of the virtual agent” (friendly vs. unfriendly), and “level of social anxiety.” For this purpose, we examined the effects on the time spent in the different rooms of the virtual environment as well as on the minimal interpersonal distance between the participants and the virtual agents. A significant interaction between “phase” and the “virtual agents’ behavior” would indicate that the conditioning influenced participants’ social behavior. Notably, for Experiment 2, where agents were moving around freely, we could not compare participants’ behavior across phases. Therefore, we only focused on the social approach–avoidance test and computed LMMs including the fixed effects “behavior of the virtual agent” (friendly vs. unfriendly) and “level of social anxiety.” As participants might have sometimes not been aware of the virtual agents approaching from behind, we also performed an exploratory analysis only including phases where the agents were actually visible to the participants.

We also analyzed effects on the average heart rate, skin conductance level, and pupil diameter recorded during the different experimental phases. Therefore, we again computed LMMs with the fixed effects “phase” (habituation vs. social approach–avoidance test), “behavior of the virtual agent” (friendly vs. unfriendly), and “level of social anxiety.” The outcomes of these analyses did not yield significant results and are reported in the Supplementary Results (https://osf.io/jmhfa).

All figures show box plots unless otherwise noted, with boxes extending from the first quartile (25%) to the third quartile (75%) of the data, with a dashed line at the median. The whiskers cover 95% of the data, with their ends lying at the 2.5th and 97.5th percentiles. Embedded gray squares show the mean, and the gray error bars visualize the upper and lower boundaries of 95% bootstrapped confidence intervals (5000 iterations) of the mean.

## Results

### Subjective ratings

**Fig. 2:**
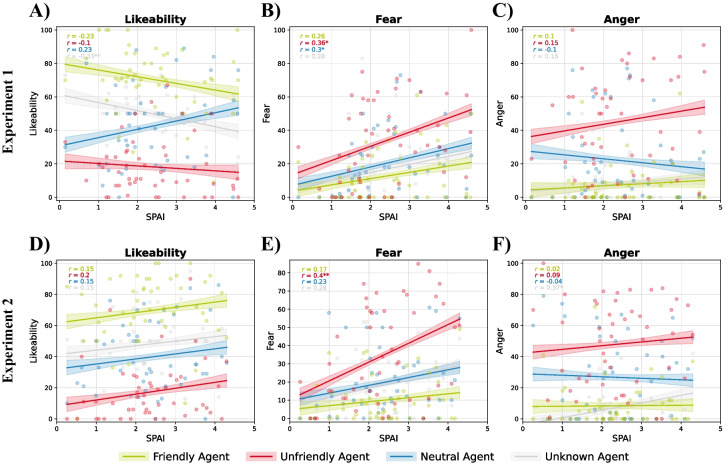
Effect of social anxiety on the subjective ratings of likeability (**A, D**), fear (**B, E**), and anger (**C, F**) for Experiment 1 (**A–C**) and Experiment 2 (**D–F**). Colored ribbons denote standard errors of the mean. Correlations between social anxiety and subjective ratings are reported separately for the different conditions as Pearson’s correlation coefficients

In both experiments, we found significant differences in the rating of the friendly and the unfriendly virtual agents regarding likeability, fear, and anger (likeability in Experiment 1: *F*(1, 46) = 209.54, *p* < .001, η_p_^2^ = .81 and in Experiment 2: *F*(1, 46) = 177.85, *p* < .001, η_p_^2^ = .79; fear in Experiment 1: *F*(1, 46) = 48.04, *p* < .001, η_p_^2^ = .50 and in Experiment 2: *F*(1, 46) = 55.08, *p* < .001, η_p_^2^ = .53; anger in Experiment 1: *F*(1, 46) = 98.14, *p* < .001, η_p_^2^ = .67 and in Experiment 2: *F*(1, 46) = 99.83, *p* < .001, η_p_^2^ = .68; see Fig. 2A–F). Similar differences were found for the ratings of attractiveness and the previous behavior of the virtual agents (see Table S1 and Fig. S8). Trait social anxiety had a significant impact on fear ratings (Experiment 1: *F*(1, 46) = 7.16, *p* = .010, η_p_^2^ = .13, see Fig. 2B; Experiment 2: *F*(1, 46) = 7.72, *p* = .008, η_p_^2^ = .14, see Fig. 2E), with socially anxious participants generally reporting more fear towards all virtual agents. In Experiment 2, we additionally observed a significant interaction of trait social anxiety and condition on fear ratings, *F*(1, 46) = 6.17, *p* = .017, η_p_^2^ = .11, with socially anxious participants indicating higher fear ratings particularly for the negatively conditioned virtual agent (see Fig. 2E). All other main and interaction effects were not statistically significant (see Table S1).

### Acquisition phase

#### Physiology

**Fig. 3:**
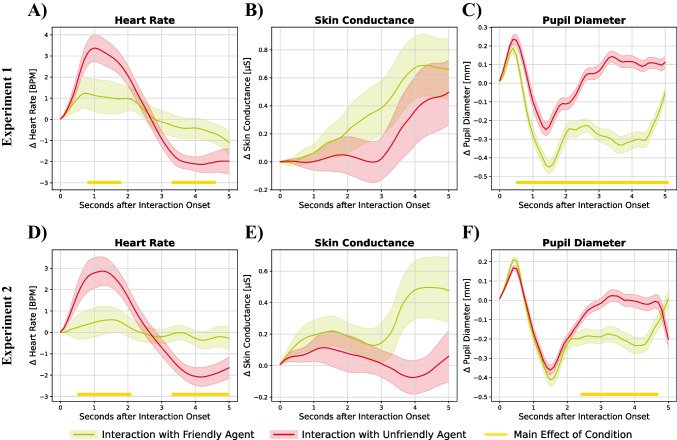
Effect of social anxiety and behavior of the virtual agent on heart rate (**A, D**), skin conductance (**B, E**), and pupil diameter (**C, F**) for Experiment 1 (**A–C**) and Experiment 2 (**D–F**). Heart rate: *N*_1_ = 42, *N*_2_ = 42; skin conductance response: *N*_1_ = 37, *N*_*2*_ = 41; pupil diameter: *N*_1_ = 48, *N*_2_ = 48. Colored ribbons denote standard errors of the mean. The yellow bar at the bottom shows a significant main effect of the condition (i.e., friendly vs. unfriendly interaction)

In the acquisition phase of Experiment 1, participants exhibited a significant biphasic heart rate pattern consisting of an initial acceleration and a subsequent deceleration when confronted with the unfriendly reaction of the negatively conditioned agent (see Fig. 3A). Accordingly, a nonparametric cluster-based permutation test indicated a significant main effect of condition (*p* < .05) for these two clusters. We did not observe significant clusters for either the main effect of social anxiety or the interaction of condition and social anxiety in heart rate responses. While there were no significant differences in skin conductance (see Fig. 3B), participants showed an increased pupil diameter following the unfriendly compared to the friendly reaction as indicated by a significant main effect of condition for a cluster spanning almost the whole interaction time (see Fig. 3C). Similar to the effects on heart rate, we did not observe statistically significant effects of social anxiety or an interaction of condition and social anxiety. These effects could be fully replicated in Experiment 2: While we observed a main effect of condition on heart rate (see Fig. 3D) and pupillary responses (see Fig. 3F), we again failed to reveal main or interaction effects concerning social anxiety. No significant effects were found for skin conductance changes (see Fig. 3E).

#### Gaze

**Fig. 4:**
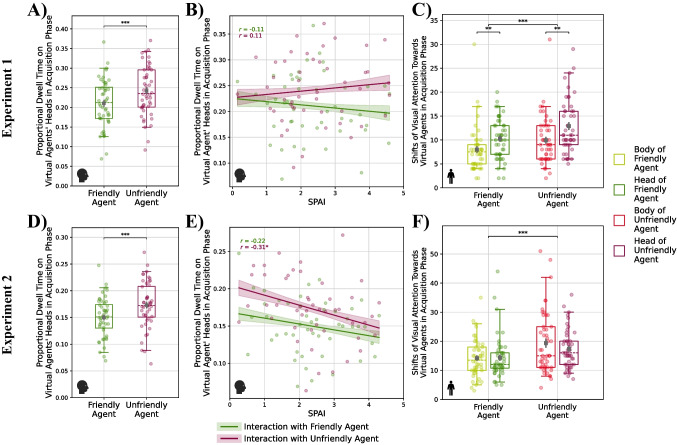
Effect of social anxiety and behavior of the virtual agent on the proportional dwell time on the virtual agents’ face/head during the acquisition phase (**A, D**), with accounting for effects of social anxiety (**B, E**) and shifts in visual attention towards the virtual agents (**C, F**) for Experiment 1 (**A–C**) and Experiment 2 (**D–F**). Brackets denote significant differences between the conditions. Colored ribbons depict standard errors of the mean. Correlations between social anxiety and the proportional dwell time on the virtual agents’ heads are reported separately for the different conditions as Pearson’s correlation coefficients

Regarding participants’ gaze behavior in the acquisition phase, analyses revealed a main effect of the region of interest in both experiments (Experiment 1: *F*(1, 184) = 254.71, *p* < .001, η_p_^2^ = .58, Experiment 2: *F*(1, 184) = 286.84, *p* < .001, η_p_^2^ = .61), indicating that during the interactions, participants focused predominantly on the virtual agents’ faces/heads rather than their bodies. Additionally, there was a main effect of the condition (Experiment 1: *F*(1, 184) = 9.02, *p* = .003, η_p_^2^ = .05, Experiment 2: *F*(1, 184) = 12.66, *p* < .001, η_p_^2^ = .06). Participants looked longer towards the unfriendly agent, particularly his face, than towards the friendly agent (see Fig. 4A and D). While in Experiment 1, dwell time was unaffected by social anxiety (see Fig. 4B), in Experiment 2, higher social anxiety was associated with less proportional dwell time on the virtual agents’ faces/heads (see Fig. 4E). All other main and interaction effects were not statistically significant (see Table S2).

As a measure of hyperscanning, we calculated how often participants redirected their attention towards a social stimulus (body or face of virtual agent). The difference between conditions was significant for both experiments (Experiment 1: *F*(1, 138) = 15.21, *p* < .001, η_p_^2^ = .10, Experiment 2: *F*(1, 138) = 24.29, *p* < .001, η_p_^2^ = .15). During the acquisition phase, participants shifted their attention more frequently towards the unfriendly virtual agent (see Fig. 4C and F). Additionally, we also observed a significant main effect of the region of interest in Experiment 1, *F*(1, 138) = 19.96, *p* < .001, η_p_^2^ = .12. Participants showed more fixation switches towards the agents’ faces/heads than towards their bodies during the interactions in the acquisition phase. The remaining main or interaction effects were not statistically significant (see Table S3).

### Test phase

#### Gaze

**Fig. 5:**
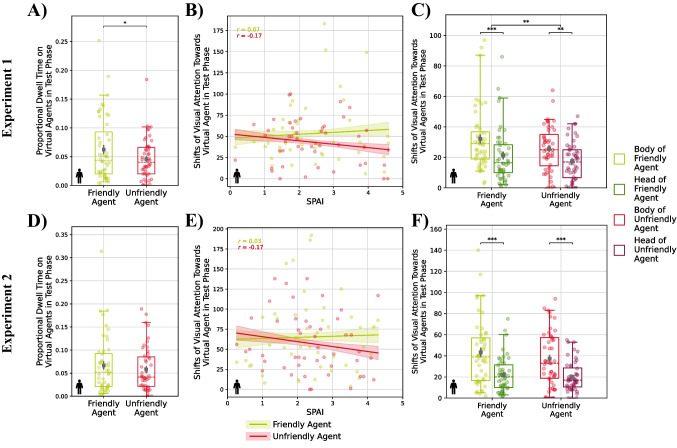
Effect of social anxiety and behavior of the virtual agent on the proportional dwell time on the virtual agents’ face/head during the test phase (**A, D**), shifts in visual attention towards the virtual agents (**B, E**) with accounting for effects of social anxiety (**C, F**) for Experiment 1 (**A–C**) and Experiment 2 (**D–F**). Brackets denote significant differences between the conditions. Colored ribbons depict standard errors of the mean. Correlations between social anxiety and the number of shifts in attention are reported separately for the different conditions as Pearson’s correlation coefficients

In contrast to the acquisition phase, participants looked significantly longer at the friendly than the unfriendly agent in the test phase of the first experiment, *F*(1, 138) = 5.86, *p* = .017, η_p_^2^ = .04 (see Fig. 5A). Likewise, they also shifted their attention more often towards the friendly than the unfriendly virtual agent (see Fig. 5C). The latter tendency was especially prevalent in socially anxious participants, as indicated by an almost significant interaction of trait social anxiety and condition, *F*(1, 138) = 3.88, *p* = .051, η_p_^2^ = .03 (see Fig. 5B). Additionally, we again observed a significant main effect of the region of interest for our measure of hyperscanning, *F*(1, 138) = 30.55, *p* < .001, η_p_^2^ = .18. However, in contrast to the acquisition phase, participants now showed more shifts in visual attention towards the virtual agents’ bodies than towards their faces/heads (see Fig. 5C).

In the second experiment, we could not replicate the main effect of condition on dwell times, *F*(1, 138) = 1.28, *p* = .259, η_p_^2^ = .01 (see Fig. 5D), and for our measure of hyperscanning, analyses only revealed a significant main effect of the region of interest in the test phase, *F*(1, 138) = 53.08, *p* < .001, η_p_^2^ = .27, where, like in the first experiment, participants exhibited more switches towards the agents’ bodies than towards their faces/heads (see Fig. 5F). All other main and interaction effects were not statistically significant (see Tables S4 and S5).

#### Behavior

**Fig. 6:**
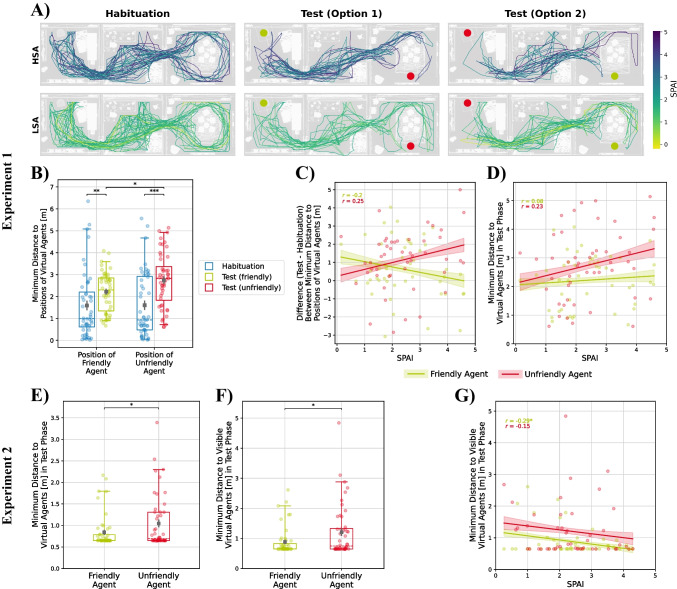
Effect of social anxiety and condition on the participants’ movement trajectories during the habituation and the test phase of Experiment 1 (**A**). The upper row comprises the subgroup of participants scoring above the median in social anxiety (as measured by the SPAI); the lower row comprises participants scoring below the median. *N*_HSA_ = 24, *N*_LSA_ = 24. The colored circles denote the position of the virtual agents in the test phase, with the green circle indicating the position of the friendly agent and red circle showing the position of the unfriendly agent. Since the position was counterbalanced between subjects, there are two options. Half of the participants experienced option 1, the other half option 2. Minimal interpersonal distance from the positions of the virtual agents in the habituation and test phase (**B**) and effect of social anxiety on differences between the minimal interpersonal distance from the positions of the virtual agents in the habituation and test phase (**C**), with positive values indicating an increased distance in the test phase and negative values indicating a decreased distance in the test phase. Minimal interpersonal distance between the participants and the virtual agents during the test phase accounting for effects of social anxiety (**D**). Minimal interpersonal distance between the participants and the virtual agents during the test phase during the test phase of Experiment 2 (**E**), when only taking into account phases where the virtual agents were in the participants’ field of view (**F**) and with accounting for effects of social anxiety (**G**). Brackets denote significant differences between the conditions. Colored ribbons depict standard errors of the mean. Correlations between social anxiety and distance scores are reported separately for the different conditions as Pearson’s correlation coefficients

In Experiment 1, we observed a significant main effect of phase on the minimal interpersonal distance, *F*(1, 138) = 30.75, *p* < .001, η_p_^2^ = .18. All participants kept greater minimal distances from the positions of the virtual agents in the test phase compared to the distance they had kept during the habituation phase when no agent was sitting at this location (see Fig. 6A and B). Importantly, we also observed a significant interaction effect involving phase, condition, and trait social anxiety, *F*(1, 138) = 5.09, *p* = .026, η_p_^2^ = .04, which indicates that in the test phase, participants with high social anxiety maintained larger minimal interpersonal distances from the unfriendly agent than the friendly agent (see Fig. 6C and D). Similarly, we observed an effect of the agents’ previous behavior on the minimal distance that participants maintained during the test phase of Experiment 2, *F*(1, 46) = 4.44, *p* = .041, η_p_^2^ = .08. After the social conditioning procedure, participants kept a greater minimal distance from the unfriendly agent than from the friendly one (see Fig. 6E). This effect was slightly stronger when only taking into account phases where the virtual agents were in the participants’ field of view, *F*(1, 46) = 5.66, *p* = .022, η_p_^2^ = .11 (see Fig. 6F). Although participants with higher social anxiety seemed to show a smaller minimal distance from both agents (see Fig. 6G), the main effect of social anxiety failed to reach statistical significance, *F*(1, 46) = 3.16, *p* = .082, η_p_^2^ = .06, as did all other main or interaction effects (see Table S6).

In both experiments, we failed to observe effects of the social conditioning procedure on the time participants spent in the same room with the different virtual agents (see Fig. S9A–D). In Experiment 2, we extended this investigation to consider the effect of the conditioning on how long the virtual agents remained within the participants’ field of view. Although there was a tendency for the friendly agent to be present in participants’ field of view for a longer duration than the unfriendly agent (see Fig. S10E), this difference failed to reach significance, *F*(1, 91) = 3.17, *p* = .078, η_p_^2^ = .03. There was no main or interaction effect involving trait social anxiety on the time spent in the same room with a virtual agent or on the time participants had a specific virtual agent in their field of view (see Table S7 and Fig. S9).

In Experiment 1, we exploratorily investigated further interaction attempts during the test phase. A significant main effect of the condition, *F*(1, 46) = 11.70, *p* = .001, η_p_^2^ = .20, indicated that participants tried to interact significantly more often with the friendly agent than the unfriendly agent (see Fig. S10). We observed no significant main effect of social anxiety on interaction attempts or an interaction between social anxiety and condition (see Table S8).

## Discussion

In the current study, we implemented a novel immersive VR scenario to investigate approach and avoidance tendencies following a one-time interaction with virtual agents in individuals differing in trait social anxiety. In two experiments, we explored approach–avoidance tendencies on a behavioral level (whole-body movement, interpersonal distance) regarding active exploration (gaze behavior) as well as on a subjective level (subjectively perceived likeability, fear, and anger). We also compared autonomic responses (pupillary, electrodermal and cardiovascular responses) during the interaction and a subsequent test phase. The difference between the two experiments lay in the behavior of the virtual agents during the social approach–avoidance test: While in Experiment 1 the agents were positioned stationary, i.e., they remained seated in one of the rooms, they dynamically changed their position by walking around in Experiment 2.

In line with our hypotheses, participants in both experiments rated the negatively conditioned virtual agent as the least likeable and the positively conditioned virtual agent as the most likeable. Participants also reported more negative emotions, i.e., fear and anger, towards the negatively conditioned virtual agent than towards the neutral and positively conditioned agents. Hence, participants who accurately recalled whether the virtual agents’ previous behavior was comparably friendly or unfriendly (an inclusion criterion) adjusted their perceived likeability, fear, and anger towards these virtual agents. In contrast to previous studies that included multiple interactions (e.g., Lange & Pauli, 2019; Lissek et al., 2008; Reichenberger et al., 2020), it is important to note that these results relied on a single interaction with the virtual agent in the current experiments. While participants generally showed rapid learning from the social interactions on a subjective level, we additionally observed a significant influence of trait social anxiety on fear ratings. Participants with higher social anxiety reported increased levels of experienced fear towards all virtual agents (both experiments), with even higher ratings for the negatively conditioned virtual agent (Experiment 2). These results fit with previous findings (Reichenberger et al., 2020) and suggest that individuals with social anxiety may exhibit heightened sensitivity to social cues and a tendency to overanalyze social situations. For them, social situations are very volatile, and heightened awareness may lead them to process and learn from social interactions more quickly to be able to adapt to social environments (Beltzer et al., 2019). However, they may also struggle with biased learning and overgeneralization, where they apply negative judgments or expectations to a broad range of social situations which could then, consistent with the current study, lead to increased fear of all agents (Zabag et al., 2022).

Apart from the subjective ratings, this bias was also evident in approach–avoidance behavior. In particular, the whole-body movement patterns demonstrated that after the interaction, participants of both experiments maintained a greater distance from the unfriendly virtual agent than the friendly one. In Experiment 1, this effect was most prevalent in participants reporting high trait social anxiety, as they seemed to be more cautious in approaching the negatively conditioned virtual agent. In terms of the time participants spent in the same room with the virtual agents, we observed a nonsignificant trend suggesting that individuals with high social anxiety tended to favor spending time in the room with the friendly virtual agent (Experiment 1). Additionally, we also found indications of increased avoidance in participants’ allocation of visual attention. Participants dwelled more on the friendly agent and reoriented their attention more frequently towards the friendly agent (Experiment 1). In other words, they avoided looking at the unfriendly one.

In particular, the findings of the first experiment are in line with our hypothesis that social anxiety is associated with greater avoidance of social threats. Socially anxious people tend to be hyperaware of threatening social cues (McTeague et al., 2018; Wermes et al., 2018), and they often interpret unfriendly behavior of others as directed towards them personally. The resulting assumption that their interaction partners hold negative opinions about them often leads to considerably lower self-esteem (Chen et al., 2020a; Iancu et al., 2015). Hence, avoiding social situations is a way to shield themselves from perceived social threats, rendering it a coping strategy to protect their self-image (Piccirillo et al., 2016). Supporting this notion, previous studies also found associations between social anxiety traits and interpersonal distance (e.g., Kroczek et al., 2020; Lange & Pauli, 2019; Rinck et al., 2010; Wieser et al., 2010). Interestingly, this relationship was absent in our Experiment 2, where virtual agents were moving around dynamically. In principle, this pattern could result from different behavioral tendencies in participants with high vs. low social anxiety. While the first group may have exhibited behavioral inhibition as a component of their fear response (Hashemi et al., 2021) upon encountering the negatively conditioned agent in their line of sight, the second group may have simply disregarded the virtual agents, showing indifference to their presence. Also, the dynamic movement of the agents might have rendered participants unable to avoid looking at the unfriendly agent as much as they desired, given his frequent appearances in their field of view (cf. Kroczek et al., 2020).

While avoidant patterns predominated in the test phase, we observed longer dwell times on the unfriendly agent in the acquisition phase of both experiments, particularly on his face/head. This behavior fits with the hypervigilance hypothesis postulating that in threatful social situations, people engage in an excessive and intense monitoring of social cues, such as others’ facial expressions and body language, to quickly identify potential negative judgment to be able to adapt the own behavior accordingly (Richards et al., 2014). However, we could not robustly link this effect to trait social anxiety in the current study. In Experiment 1, we observed no significant impact of social anxiety on measures of visual exploration. Intriguingly, in Experiment 2, a negative relationship emerged between social anxiety levels and attention directed at both agents. This indicated that socially anxious individuals rather showed unselected gaze aversion instead of hypervigilance. While the vigilance–avoidance theory primarily aims to characterize behavior in anxiety disorders (Chen & Clarke, 2017; Richards et al., 2014), some elements of the proposed behavioral pattern can also be observed in non-pathological coping mechanisms, albeit in a less intense or pervasive form. In everyday life, people might show transient vigilance to potential threats, because such behaviors can be adaptive, helping individuals to assess and respond to potential dangers (Eilam et al., 2011; Gomes & Semin, 2020).

This stage of heightened sensory awareness is often accompanied by threat-specific autonomic reactions, which we also observed during the acquisition phase in the current study. In both experiments, there was an initial acceleration in heart rate followed by a subsequent deceleration (bradycardia) in response to the unfriendly agent’s approach and subsequent insult. The decelerative component in particular has been discussed as a typical defensive reaction to threatening cues (Bradley et al., 2001), which could also be interpreted as some kind of “freezing” response induced by the rude reaction of the unfriendly agent (Roelofs, 2017). In addition to the cardiac response, participants exhibited stronger pupil dilation during the negative interaction in both experiments. This “threat-inhibited pupillary light response” allows more light to enter the eyes and thereby enhances visual processing in preparation for an adequate threat response (Bitsios et al., 2004; Ojala & Bach, 2020). Taken together, the autonomic reactions in heart rate and pupil diameter indicate a co-activation of the sympathetic and parasympathetic branches of the autonomic nervous system which characterize a state of attentive immobility (Merscher et al., 2022; Roelofs, 2017; Volchan et al., 2017). It can be observed in animals and humans, serves as an early orienting response, facilitates the detection of relevant information, and ultimately helps to identify appropriate actions to deal with a stressor, i.e., a subsequent fight-or-flight response (Fanselow & Lester, 1988; Noordewier et al., 2020; Roelofs et al., 2010; Roelofs & Dayan, 2022).

It is noteworthy that prior similar studies have also reported variations in skin conductance as an indicator of threat (Kroczek et al., 2020; Reichenberger et al., 2017), which were not observed in the current study. Nonetheless, these studies differ from our study in two key aspects. First, they incorporated a higher number of acquisition trials at the individual level, thereby enhancing statistical power for comparing different conditions. Second, it is crucial to recognize that skin conductance responses are not exclusively tied to threat; they can also manifest as reactions to other emotional stimuli, such as in response to a friendly and engaging interaction (Andreatta & Pauli, 2015; Ojala & Bach, 2020).

To summarize, our multimodal study revealed that individuals initially exhibit vigilance to social threats followed by subsequent avoidance of the offender. Individual differences in social anxiety were related only to aspects of avoidance behavior and not to hypervigilance. While there is generally some debate about whether and to what extent the vigilance–avoidance hypothesis can explain behavioral patterns in social anxiety, recent systematic reviews and meta-analyses have highlighted the influence of the experimental methods and the type of stimuli used (Chen et al., 2020b; Chen & Clarke, 2017; Günther et al., 2021). The common conclusion of these studies is that as the situational context becomes more naturalistic, individuals are increasingly prone to engage in avoidance rather than displaying hypervigilance. Since the current experimental scenario was explicitly designed to increase ecological validity, it is not surprising that the avoidance pattern predominated in anxious participants. Further research is needed to explore the influence of the experimental design and the stimuli on the behavioral patterns and underlying neural processes elicited by the confrontation with (social) threats. Additionally, further study of how trait anxiety modulates these responses is critical.

While the current study demonstrates the potential of immersive virtual environments for examining behavioral adaptations under realistic conditions and provides important insights into the development of adaptive approach and avoidance tendencies after single social learning experiences, some limitations should also be acknowledged: First, while the use of the omnidirectional treadmill allowed participants to move more naturally in the virtual environment than in previous studies that implemented passive movement using a joystick, gamepad, or teleportation (e.g., Gromer et al., 2021; Kroczek et al., 2020; Marusak et al., 2017; Reichenberger et al., 2017), the strenuous movement on the treadmill impaired the quality of physiological measurements. This is also a possible reason for the high number of excluded participants due to a substantial increase in motion sickness symptoms from the pre- to post-measurement.

Second, we only investigated females in the current study. Previous studies have shown that social anxiety is more prevalent in women (Asher et al., 2017; McLean et al., 2011), and especially in interactions with male counterparts (Reichenberger et al., 2019; Wieser et al., 2010). Since we aimed for robust effects to test the virtual paradigm, we hence opted for female participants in combination with male virtual agents. Recruiting a rather homogeneous sample also provides greater control of confounding variables, which is particularly beneficial when employing and validating a novel experimental paradigm such as ours. However, to ensure that the current results also generalize to males, future studies should aim to replicate the effects in more heterogeneous samples. Additionally, our sample mainly consisted of university students, who due to their age and educational background might be more technologically proficient or familiar with VR than the general public. This fact could affect the presence and immersion in the virtual scenario and thereby influence the effects elicited by the virtual interactions. Although there is initial research exploring age differences in presence and cybersickness (Dilanchian et al., 2021; Huygelier et al., 2019), further, more targeted investigation focusing in particular on older adults is necessary to evaluate the generalizability of our findings.

Third, it is important to note that while in both studies we were able to recruit participants with a wide range of social anxiety symptoms, spanning from low to medium levels and extending to (sub)clinical manifestations, our study did not include individuals with a confirmed clinical diagnosis of social anxiety disorder. Consistent with a dimensional conceptualization of psychopathology (Cuthbert, 2014; Kozak & Cuthbert, 2016), we thus focused on continuous measures of trait social anxiety instead of separating participants into healthy and clinical groups. This approach also allows us to investigate how certain maladaptive behaviors may function as markers for psychopathology rather than merely representing its sequelae (Morriss et al., 2021; Wong et al., 2023). Since results might differ in clinically diagnosed samples, we recommend evaluating the stability and generalizability of the current findings in individuals formally diagnosed with social anxiety disorders.

In general, we are optimistic about the potential of VR as a promising avenue for studying social learning processes and for treating social anxiety. The capacity of VR to create naturalistic, controlled, and customizable environments that closely mirror key aspects of real-life social interactions makes it a valuable tool for conducting ecologically valid research and implementing therapeutic applications with realistic exposure situations (Patotskaya et al., 2023). While we acknowledge that VR-based experiments may currently require specialized expertise and resources, the increasing accessibility of VR platforms and common standards for VR hardware and software like *OpenXR* hold promise for enabling broader adoption, exchange of implemented paradigms between labs and, ultimately, direct replication of experiments in the future. A prominent example for an openly available and flexible toolkit for creating VR experiments that can be shared across research labs to facilitate replication is the “VRthreat toolkit for Unity” developed by Brookes and colleagues (2024). The rapid advancements in VR and related technologies, such as the integration of large language models (LLMs) and embodied agents, provide exciting opportunities for the innovative design and implementation of experiments in social neuroscience (Kroczek et al., 2024). These innovations have the potential to make interactions with virtual agents even more dynamic, interactive, and realistic, further enhancing the ecological validity of such studies. Naturally, the pace of these technological developments also presents a challenge to researchers, as the tools and paradigms used in current VR studies may become quickly outdated. While this is an inherent risk of working with cutting-edge technology, it also underscores the importance of openly sharing paradigms and frameworks to ensure that they remain useful as foundational resources for future advancements in the field.

Finally, one could consider the one-off nature of the interaction a limitation of the current study, as it contrasts with the repeated trials or longitudinal designs often used in conditioning research (Lonsdorf et al., 2017). While this may seem restrictive, however, it is important to note that in daily life, individuals regularly have to learn from single encounters or interactions, making our design an ecologically valid approach that mirrors real-world scenarios where immediate learning is critical. However, to investigate more stable and long-lasting behavioral tendencies, especially in the context of maladaptive avoidance behaviors and psychopathology, future research could benefit from employing designs that involve multiple exposures or interactions. Such approaches may be particularly relevant when studying how social anxiety develops and persists over time, as repeated interactions could provide deeper insights into the mechanisms underlying these behaviors and their resistance to change (Åhs et al., 2017). Additionally, incorporating extinction or reversal learning paradigms into social VR contexts would allow researchers to examine the flexibility of social learning processes. This could reveal how individuals adapt to changes in social contingencies, which is particularly relevant for understanding the rigidity of avoidance behaviors often associated with social anxiety (Zabag et al., 2024).

In summary, the current study demonstrated that humans adapt their subjective perception of another (virtual) person as well as their social approach and avoidance behavior promptly after having just one social learning experience. Furthermore, these processes were influenced by individual differences in trait social anxiety. The currently developed VR scenario can be seen as a basis for future work studying how variability in social interactions impacts behavior and physiological adaptations using a controlled yet dynamic manipulation of social environments while maintaining ecological validity (Lemmens et al., 2021).

## Supplementary Information

Below is the link to the electronic supplementary material. 

Supplementary file1 (1.55 MB)

## Data Availability

A demonstration video, supplementary material, and aggregated data are openly available on the Open Science Framework (OSF) at https://osf.io/mc85y/. The two VR games and raw data are available from the lead contact upon request.

## Code availability

The code for all analyses and plots is openly available on the Open Science Framework (OSF) at https://osf.io/mc85y/.
