# Supplementary material for: Studying the influence of single social interactions on approach and avoidance behavior: A multimodal investigation in immersive virtual reality
Source: Behav Res Methods. 2025 Apr 25;57(6):157. doi: 10.3758/s13428-025-02627-0 (PMC12031922; doi:10.3758/s13428-025-02627-0)
Supplement: Supplementary file 1 — Supplementary file1 (1.55 MB) [file 13428_2025_2627_MOESM1_ESM.pdf]

Supplementary Materials for
"Studying the Influence of Single Social Interactions on Approach and Avoidance Behavior – A Multimodal Investigation in Immersive Virtual Reality"

Sabrina Gado[1, [0000-0002-9792-0895]] and Matthias Gamer[1, [0000-0002-9676-9038]]

Supplementary Methods 1
- Distribution of Social Anxiety Traits 1
- Omnidirectional Treadmill 1
- Eye-Tracking Validation in VR 2
- Floor Plan 3
- Questionnaires 4
  - Simulator Sickness 5
  - Presence 6

Supplementary Results 7
- Ratings 7
- Acquisition Phase 9
  - Gaze 9
- Test Phase 11
  - Gaze 11
  - Behavior 13
  - Physiology 17

References 21

Supplementary Methods

Distribution of Social Anxiety Traits

Figure S1

Range of Social Anxiety Traits

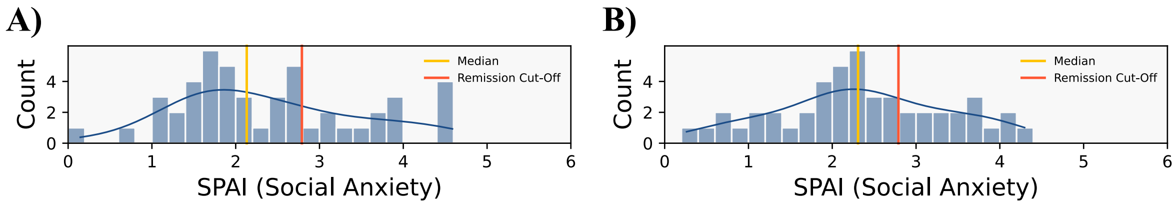

Note. Range of social anxiety traits for Experiment 1 (A) and Experiment 2 (B). The remission cut-off refers to a level of social anxiety where patients no longer met the criteria for an SAD diagnosis. It was determined using ROC methodology to balance sensitivity and specificity in the classification of 359 patients under treatment (von Glischinski et al., 2018).

Omnidirectional Treadmill

Figure S2

Omnidirectional Treadmill Enabling Participants to Explore the Virtual Environment With a Walking-Like Locomotion

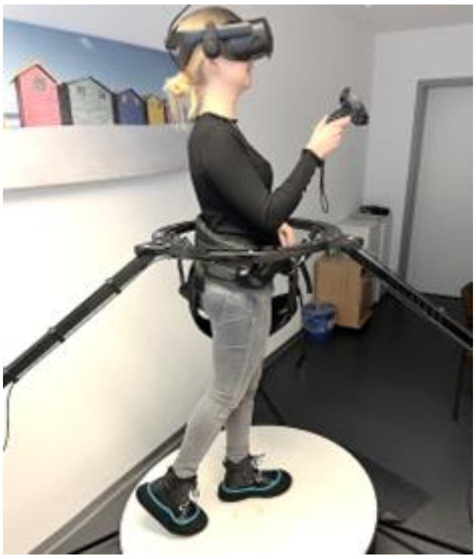

Eye-Tracking Validation in VR

Figure S3

Eye-Tracking Validation

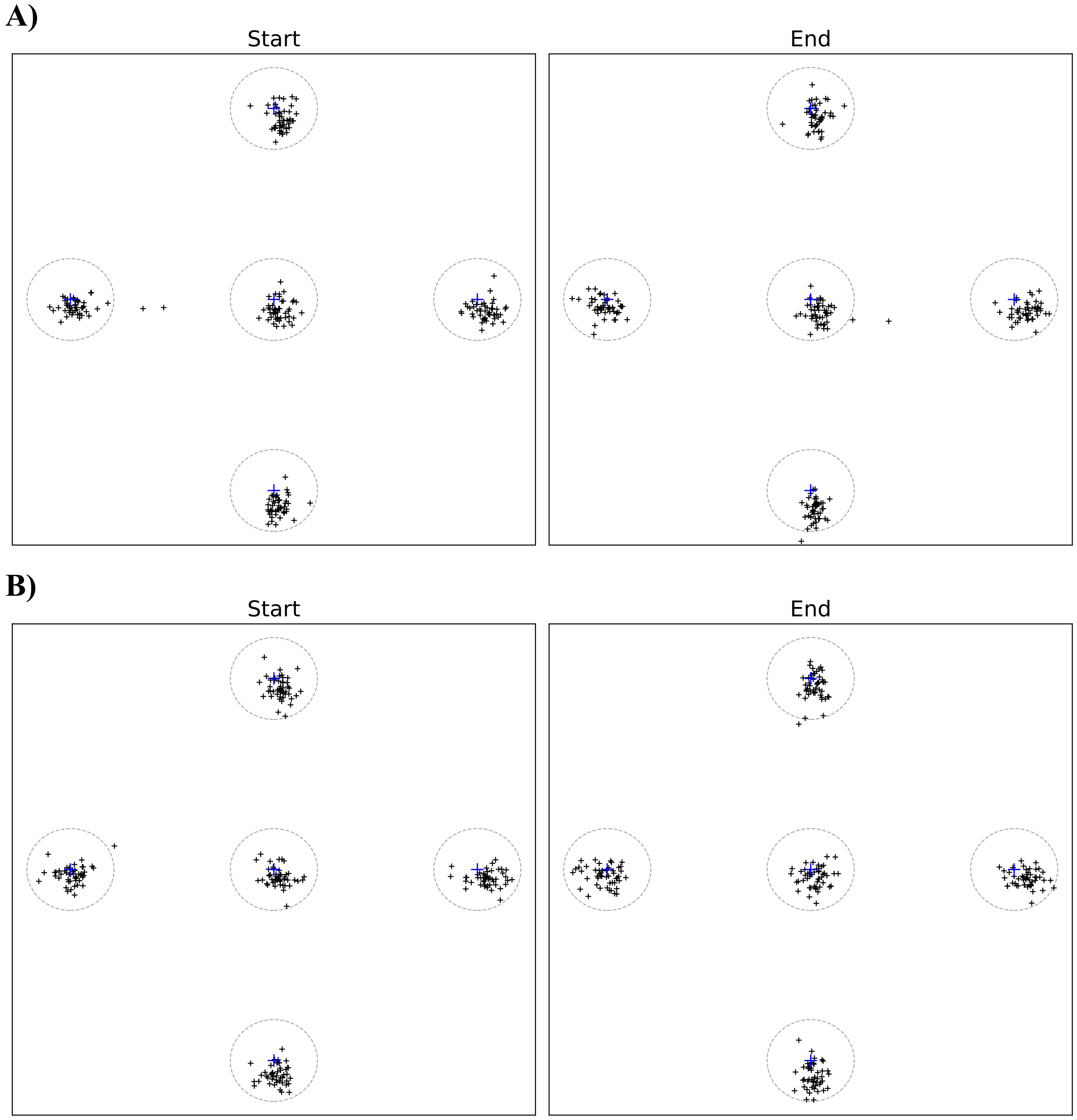

Note. Validation eye-tracking for Experiment 1 (A) and Experiment 2 (B). The blue cross represents the target point for the eye-tracking validation procedure (implemented as a black sphere with a radius of 5 cm in front of a white wall moving smoothly from one target point to the other). Participants were standing in front of the wall at a distance of 2.45 m. The target points had a maximum distance of 1.4 m. Only when the participants' gaze was within a radius of 15 cm around the center of the sphere for 500 ms (denoted by the dashed circle around the target point), the next target point was presented. The black crosses show the last recorded gaze position of participants during this 500 ms phase.

Floor Plan

Figure S4

Floor Plan

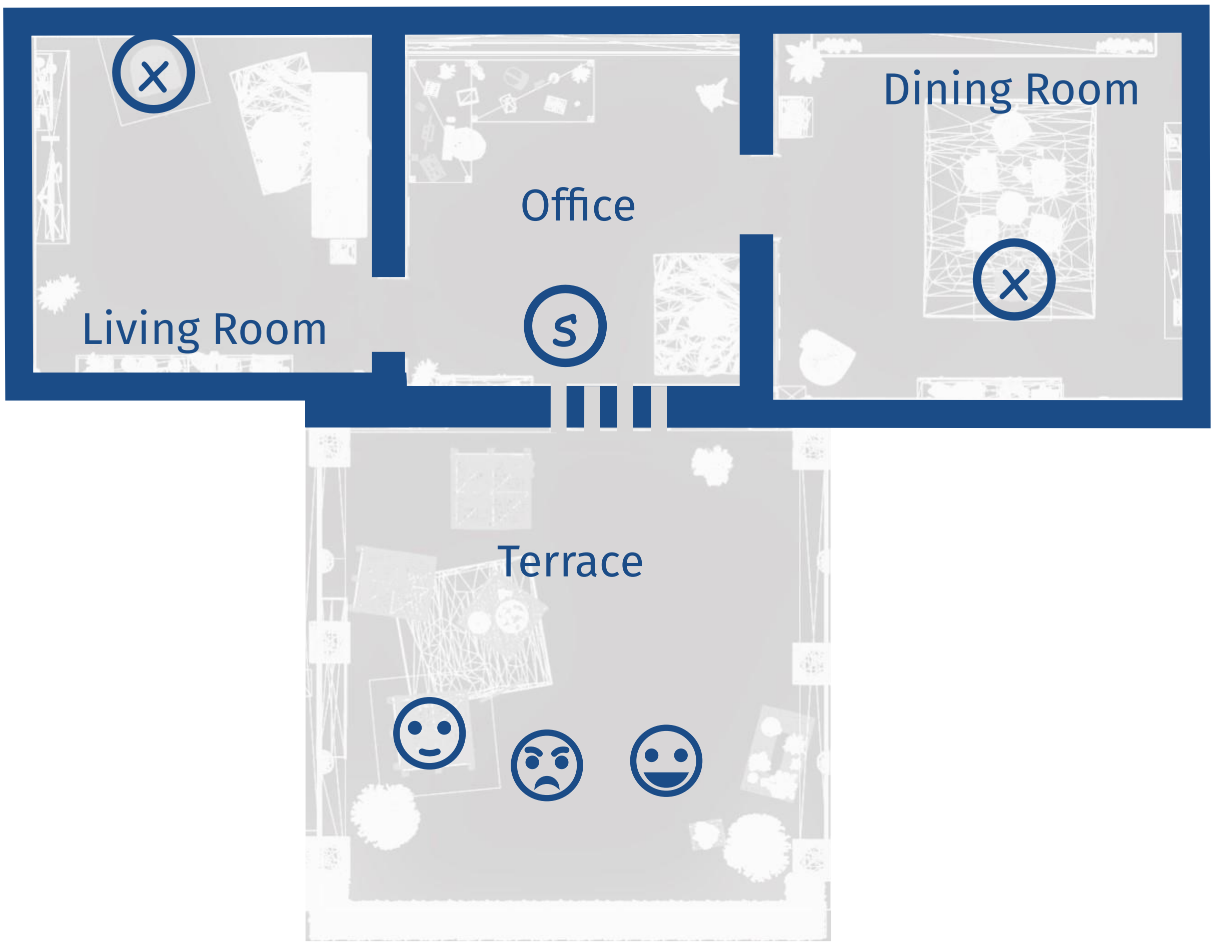

Note. The flat consisted of three rooms (living room, dining room, and office). The three rooms of the apartment were connected and freely accessible. The terrace was connected to the apartment by a closed door and could only be accessed during the acquisition phase. The “s” demarks the position where the participant was teleported at the beginning of the experiment and the start of every exploration. The “x”s demark the positions where the virtual agents were sitting during the test phase of Experiment 1. The emojis indicate where the virtual agents were placed during the acquisition phase.

Questionnaires

Participants' trait social anxiety was captured using the German version of the Social Phobia and Anxiety Inventory (SPAI; Fydrich et al., 1995) and the German version of the Social Interaction Anxiety Scale (SIAS; Stangier et al., 1999). As the SPAI is more detailed and offers slightly better discriminant validity (Peters, 2000), it was used as the primary measure of social anxiety in this study.

To assess symptoms of motion sickness, participants completed the Simulator Sickness Questionnaire (SSQ; Kennedy et al., 1993). Participants who reported a substantial increase in motion sickness symptoms from before to after the experiment were excluded from the analyses. The exclusion criterion was defined as values exceeding one standard deviation above the sample mean of the difference between post-experiment symptoms and pre-experiment symptoms, which resulted in a threshold of 38.6 (for Experiment 1, see Figure S5A) and 40.9 (for Experiment 2, see Figure S5B).

Moreover, the experienced presence was captured using the Igroup Presence Questionnaire (IPQ; Schubert et al., 2001) and the Multimodal Presence Scale (MPS; Makransky et al., 2017; Volkmann et al., 2018). Presence is defined as a "sense of being there" (Sanchez-Vives & Slater, 2005; Sheridan, 1992). When participants feel present in the virtual environment, they are more likely to engage with it, leading to increased immersion and a more authentic experience (Vasser & Aru, 2020). This can help to elicit genuine reactions and behaviors from participants. Capturing the experienced "presence" in VR experiments is crucial because it indicates the authenticity of the virtual environment, making the research more ecologically valid. The median presence ratings of 3.4 (Experiment 1, see Figure S6A) and 3.3 (Experiment 2, Figure S6B) were not particularly high but comparable to other VR environments used in scientific studies (see Melo et al., 2023).

Simulator Sickness

Figure S5

Simulator Sickness Questionnaire (Difference: Post – Pre)

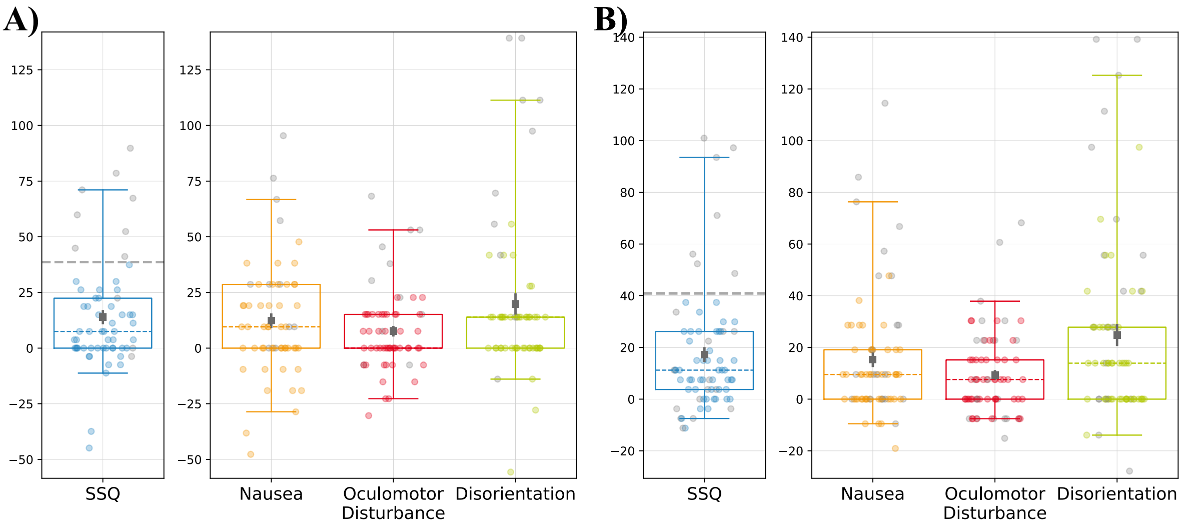

Note. The difference of the Post – Pre Values from the Simulator Sickness Questionnaire are shown for Experiment 1 (A) and Experiment 2 (B). Scores indicate the values reported after the VR phase minus the values reported before the VR phase. The dashed grey line indicates the threshold for exclusions based on the motion-sickness-criterium. Grey dots represent participants who were excluded either because a substantial increase in symptoms of motion sickness or because of forgetting or confusing the virtual agents' previous behavior.

Presence

Figure S6

Igroup Presence Questionnaire

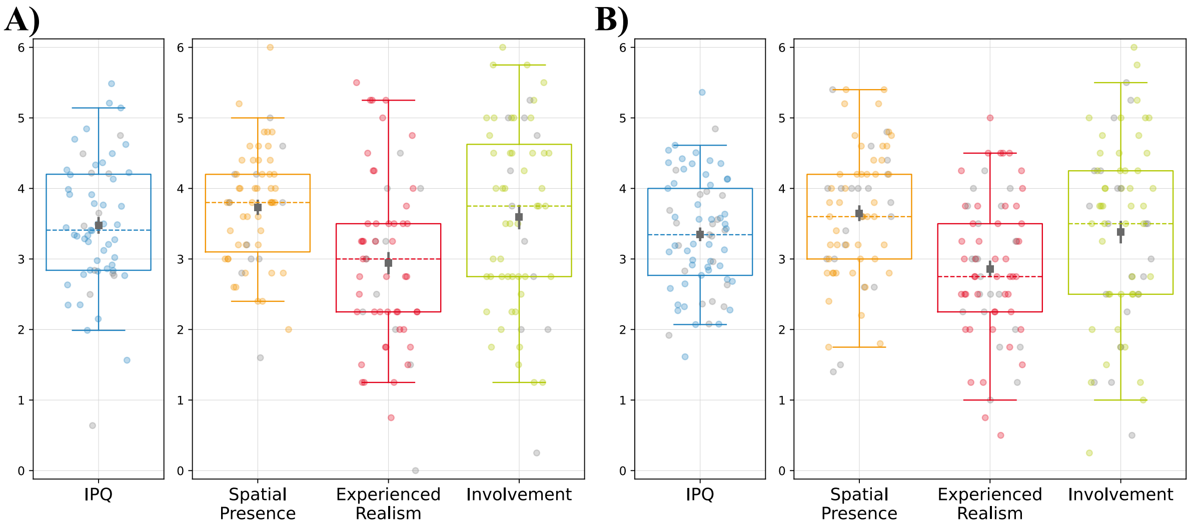

Note. Igroup Presence Questionnaire for Experiment 1 (A) and Experiment 2 (B). All items have a range from 0 to 6 with the scales being computed as mean values. Grey dots represent participants who were excluded either because of a substantial increase in symptoms of motion sickness or because of forgetting or confusing the virtual agents' previous behavior.

Figure S7

Multimodal Presence Scale

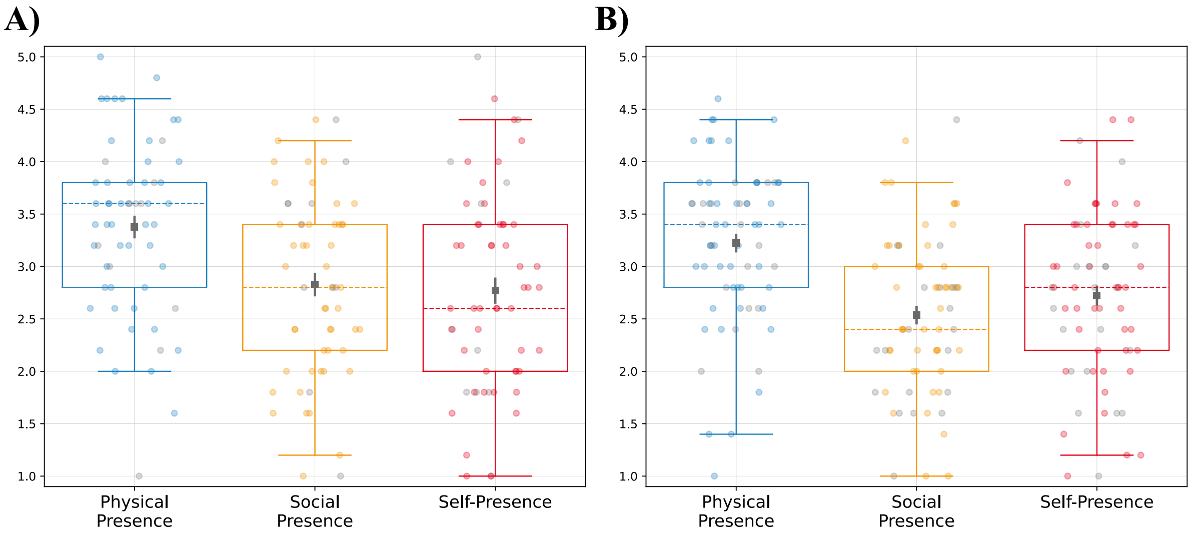

Note. Multimodal Presence Scale for Experiment 1 (A) and Experiment 2 (B). All items have a range from 1 to 5 with the scales being computed as mean values. Grey dots represent participants who were excluded either because a substantial increase in symptoms of motion sickness or because of forgetting or confusing the virtual agents' previous behavior.

Supplementary Results

Ratings

Figure S8

Subjective Ratings of Likeability, Fear, and Anger in Experiment 1 (A) and Experiment 2 (B)

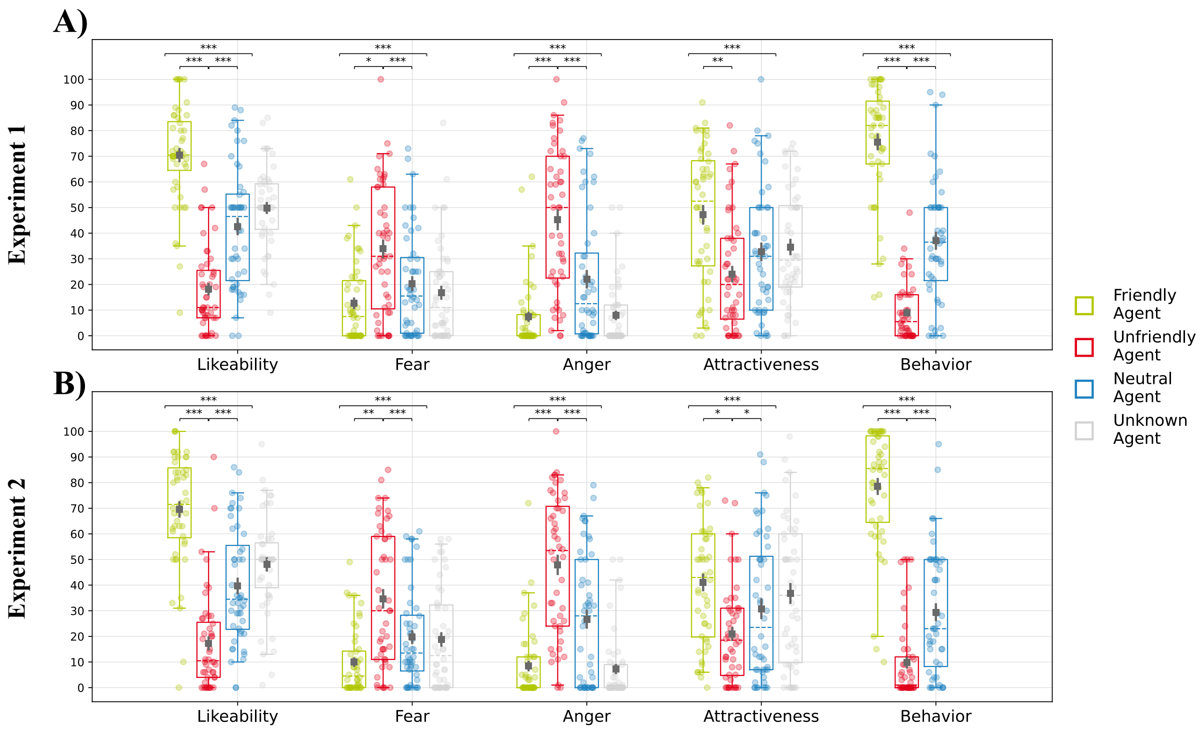

Note. Subjective ratings of likeability, fear, anger, attractiveness, and previous behavior. Brackets denote significant differences between the conditions.

Table S1

Results of the LMM With the Fixed Effects Condition and Level of Social Anxiety (SPAI) on Subjective Ratings

Factor	*F*	*df*	*p*	η_p^2	η_p^2 95% CI [LL, UL]
			**Experiment 1**		
**Likeability**					
Condition	209.54	1, 46	**< .001**	**.81**	**[.52, .95]**
SPAI	2.52	1, 46	.119	.05	[.01, .18]
Condition × SPAI	0.57	1, 46	.455	.01	[.00, .05]
**Fear**					
Condition	48.04	1, 46	**< .001**	**.50**	**[.20, .80]**
SPAI	7.16	1, 46	**.010**	**.13**	**[.04, .38]**
Condition × SPAI	2.76	1, 46	.103	.05	[.01, .19]
**Anger**					
Condition	98.14	1, 46	**< .001**	**.67**	**[.34, .89]**
SPAI	1.17	1, 46	.286	.02	[.01, .09]
Condition × SPAI	0.57	1, 46	.453	.01	[.00, .05]
**Attractiveness**					
Condition	28.47	1, 46	**< .001**	**.37**	**[.13, .71]**
SPAI	2.07	1, 46	.157	.04	[.01, .15]
Condition × SPAI	0.88	1, 46	.353	.02	[.00, .07]
**Previous Behavior**					
Condition	327.91	1, 92	**< .001**	**.78**	**[.47, .93]**
SPAI	0.00	1, 92	.971	.00	[.00, .00]
Condition × SPAI	0.01	1, 92	.943	.00	[.00, .00]
			**Experiment 2**		
**Likeability**					
Condition	177.85	1, 46	**< .001**	**.79**	**[.48, .94]**
SPAI	2.59	1, 46	.114	.05	[.01, .18]
Condition × SPAI	0.01	1, 46	.916	.00	[.00, .00]
**Fear**					
Condition	55.08	1, 46	**< .001**	**.53**	**[.22, .82]**
SPAI	7.72	1, 46	**.008**	**.14**	**[.04, .39]**
Condition × SPAI	6.17	1, 46	**.017**	**.11**	**[.03, .34]**
**Anger**					
Condition	99.83	1, 46	**< .001**	**.68**	**[.34, .89]**
SPAI	0.28	1, 46	.602	.01	[.00, .02]
Condition × SPAI	0.31	1, 46	.580	.01	[.00, .03]
**Attractiveness**					
Condition	26.42	1, 46	**< .001**	**.35**	**[.12, .69]**
SPAI	0.02	1, 46	.897	.00	[.00, .00]
Condition × SPAI	0.45	1, 46	.507	.01	[.00, .04]
**Previous Behavior**					
Condition	299.68	1, 92	**< .001**	**.76**	**[.45, .93]**
SPAI	2.38	1, 92	.126	.02	[.01, .09]
Condition × SPAI	1.16	1, 92	.284	.01	[.00, .05]

Note. SPAI = Score of the social phobia and anxiety inventory.

Acquisition Phase

Gaze

Table S2

Results of the LMM With Fixed the Effects Condition and Level of Social Anxiety (SPAI) and Region of Interest (ROI) on the Proportional Dwell Time on Virtual Agents During the Acquisition Phase

Factor	*F*	*df*	*p*	η_p^2	η_p^2 95% CI [LL, UL]
		**Experiment 1**			
Condition	9.02	1, 184	**.003**	**.05**	**[.01, .16]**
SPAI	0.00	1, 184	.958	.00	[.00, .00]
ROI	254.71	1, 184	**< .001**	**.58**	**[.26, .84]**
Condition × SPAI	0.02	1, 184	.888	.00	[.00, .00]
Condition × ROI	0.20	1, 184	.658	.00	[.00, .00]
SPAI × ROI	0.00	1, 184	.949	.00	[.00, .00]
Condition × SPAI × ROI	1.79	1, 184	.183	.01	[.00, .04]
		**Experiment 2**			
Condition	12.66	1, 184	**< .001**	**.06**	**[.02, .21]**
SPAI	4.81	1, 184	**.029**	**.03**	**[.01, .09]**
ROI	286.84	1, 184	**< .001**	**.61**	**[.28, .86]**
Condition × SPAI	0.96	1, 184	.330	.01	[.00, .02]
Condition × ROI	0.01	1, 184	.903	.00	[.00, .00]
SPAI × ROI	1.47	1, 184	.227	.01	[.00, .03]
Condition × SPAI × ROI	0.01	1, 184	.927	.00	[.00, .00]

Note. SPAI = Score of the social phobia and anxiety inventory, ROI = Region of interest (body or face/head).

Table S3

Results of the LMM With Fixed the Effects Condition and Level of Social Anxiety (SPAI) and Region of Interest (ROI) on the Shifts of Visual Attention Towards the Virtual Agents During the Acquisition Phase

Factor	*F*	*df*	*p*	η_p^2	η_p^2 95% CI [LL, UL]
**Experiment 1**					
Condition	15.21	1, 138	**< .001**	**.10**	**[.03, .30]**
SPAI	3.78	1, 46	**.058**	**.07**	**[.02, .24]**
ROI	19.96	1, 138	**< .001**	**.12**	**[.04, .36]**
Condition × SPAI	1.20	1, 138	.275	.01	[.00, .03]
Condition × ROI	0.32	1, 138	.571	.00	[.00, .01]
SPAI × ROI	0.11	1, 138	.740	.00	[.00, .00]
Condition × SPAI × ROI	0.03	1, 138	.862	.00	[.00, .00]
**Experiment 2**					
Condition	24.29	1, 138	**< .001**	**.15**	**[.04, .40]**
SPAI	0.45	1, 46	.504	.01	[.00, .04]
ROI	1.12	1, 138	.291	.01	[.00, .03]
Condition × SPAI	0.34	1, 138	.562	.00	[.00, .01]
Condition × ROI	1.59	1, 138	.210	.01	[.00, .04]
SPAI × ROI	0.96	1, 138	.329	.01	[.00, .03]
Condition × SPAI × ROI	0.25	1, 138	.616	.00	[.00, .01]

Note. SPAI = Score of the social phobia and anxiety inventory, ROI = Region of interest (body or face/head).

Test Phase

Gaze

Table S4

Results of the LMM With Fixed the Effects Condition and Level of Social Anxiety (SPAI) and Region of Interest (ROI) on the Proportional Dwell Time on Virtual Agents During the Test Phase

Factor	*F*	*df*	*p*	η_p^2	η_p^2 95% CI [LL, UL]
			**Experiment 1**		
Condition	5.86	1, 138	**.017**	**.04**	**[.01, .14]**
SPAI	0.24	1, 46	.627	.00	[.00, .02]
ROI	0.88	1, 138	.351	.01	[.00, .02]
Condition × SPAI	1.60	1, 138	.208	.01	[.00, .04]
Condition × ROI	0.34	1, 138	.559	.00	[.00, .01]
SPAI × ROI	0.02	1, 138	.884	.00	[.00, .00]
Condition × SPAI × ROI	0.15	1, 138	.698	.00	[.00, .00]
			**Experiment 2**		
Condition	1.28	1, 138	.259	.01	[.00, .03]
SPAI	0.05	1, 46	.827	.00	[.00, .00]
ROI	1.13	1, 138	.290	.01	[.00, .03]
Condition × SPAI	1.55	1, 138	.216	.01	[.00, .04]
Condition × ROI	0.08	1, 138	.781	.00	[.00, .00]
SPAI × ROI	0.03	1, 138	.856	.00	[.00, .00]
Condition × SPAI × ROI	0.01	1, 138	.938	.00	[.00, .00]

Note. SPAI = Score of the social phobia and anxiety inventory, ROI = Region of interest (body or face/head).

Table S5

Results of the LMM With Fixed the Effects Condition and Level of Social Anxiety (SPAI) and Region of Interest (ROI) on the Shifts of Visual Attention Towards the Virtual Agents During the Test Phase

Factor	*F*	*df*	*p*	η_p^2	η_p^2 95% CI [LL, UL]
**Experiment 1**					
Condition	8.95	1, 138	**.003**	**.06**	**[.02, .20]**
SPAI	0.06	1, 46	.801	.00	[.00, .01]
ROI	30.55	1, 138	< **.001**	**.18**	**[.05, .46]**
Condition × SPAI	3.88	1, 138	**.051**	**.03**	**[.01, .10]**
Condition × ROI	0.73	1, 138	.394	.01	[.00, .02]
SPAI × ROI	0.01	1, 138	.933	.00	[.00, .00]
Condition × SPAI × ROI	1.08	1, 138	.300	.01	[.00, .03]
**Experiment 2**					
Condition	2.35	1, 138	.128	.02	[.00, .06]
SPAI	0.25	1, 46	.619	.01	[.00, .02]
ROI	53.08	1, 138	< **.001**	**.27**	**[.09, .60]**
Condition × SPAI	2.10	1, 138	.150	.01	[.00, .06]
Condition × ROI	0.54	1, 138	.462	.00	[.00, .01]
SPAI × ROI	0.25	1, 138	.617	.00	[.00, .01]
Condition × SPAI × ROI	0.00	1, 138	.996	.00	[.00, .00]

Note. SPAI = Score of the social phobia and anxiety inventory, ROI = Region of interest (body or face/head).

Behavior

Table S6

Results of the LMM With Fixed the Effects Phase, Condition and Level of Social Anxiety (SPAI) on Minimum Interpersonal Distance

Factor	*F*	*df*	*p*	η_p^2	η_p^2 95% CI [LL, UL]
**Experiment 1**					
Phase	30.75	1, 138	**< .001**	**.18**	**[.05, .46]**
Condition	2.79	1, 138	.097	.02	[.01, .07]
SPAI	1.61	1, 46	.211	.03	[.01, .12]
Phase × Condition	2.58	1, 138	.111	.02	[.00, .07]
Phase × SPAI	0.07	1, 138	.787	.00	[.00, .00]
Condition × SPAI	0.91	1, 138	.343	.01	[.00, .02]
Phase × Condition × SPAI	5.09	1, 138	**.026**	**.04**	**[.01, .12]**
**Experiment 2**					
Condition	4.44	1, 46	**.041**	**.08**	**[.02, .27]**
SPAI	2.42	1, 46	.127	.05	[.01, .17]
Condition × SPAI	0.06	1, 46	.813	.00	[.00, .00]
**Experiment 2**[a]					
Condition	5.66	1, 46	**.022**	**.11**	**[.03, .32]**
SPAI	3.16	1, 46	.082	.06	[.02, .21]
Condition × SPAI	0.00	1, 46	.955	.00	[.00, .00]

Note. SPAI = Score of the social phobia and anxiety inventory.

[a] When the virtual agent was visible to the participant.

Table S7

Results of the LMM With Fixed the Effects Phase, Condition and Level of Social Anxiety (SPAI) on the Total Duration Spent in the Same Room as the Virtual Agents

Factor	*F*	*df*	*p*	η_p^2	η_p^2 95% CI [LL, UL]
**Experiment 1**					
Phase	1.11	1, 163	.294	.01	[.00, .03]
Condition	1.37	1, 163	.244	.01	[.00, .03]
SPAI	1.18	1, 163	.279	.01	[.00, .03]
Phase × Condition	0.71	1, 163	.400	.00	[.00, .02]
Phase × SPAI	0.00	1, 163	.952	.00	[.00, .00]
Condition × SPAI	0.01	1, 163	.932	.00	[.00, .00]
Phase × Condition × SPAI	1.57	1, 163	.213	.01	[.00, .04]
**Experiment 2**					
Condition	0.04	1, 90	.851	.00	[.00, .00]
SPAI	0.26	1, 90	.608	.00	[.00, .01]
Condition × SPAI	0.80	1, 90	.374	.01	[.00, .03]
**Experiment 2**[a]					
Condition	3.17	1, 91	.078	.03	[.01, .12]
SPAI	0.33	1, 91	.568	.00	[.00, .01]
Condition × SPAI	0.31	1, 91	.576	.00	[.00, .01]

Note. SPAI = Score of the social phobia and anxiety inventory.
[a] total duration a virtual agent was in participants' field of view.

Figure S9

Effect of Social Anxiety and Condition on the Total Duration Spent in the Room with the Virtual Agents

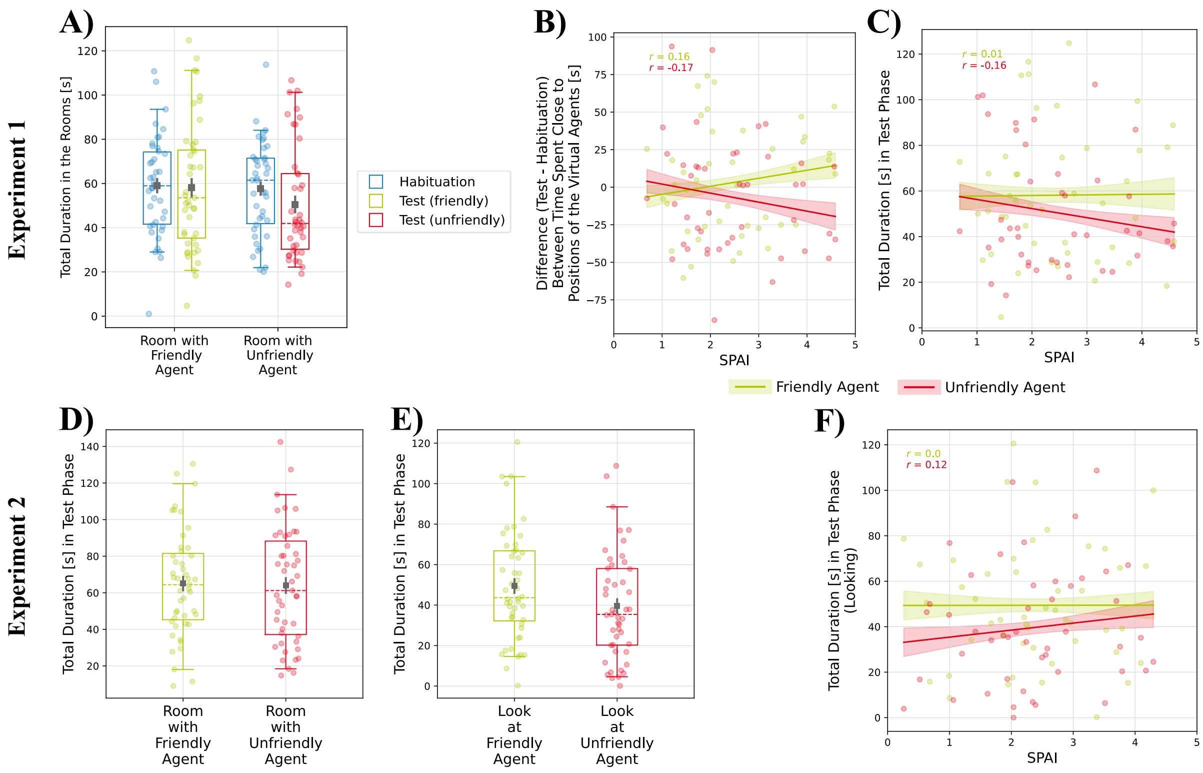

Note. Total duration spent in the room in the habituation (without virtual agents) and test phase (with virtual agents) of Experiment 1 (A) and effect of social anxiety on differences between the time spent in the room in the habituation phase and the time spent in the room in the test phase (B) with positive values indicating a longer duration and negative values indicating a shorter duration spent in the room in the test phase, respectively. Total duration spent in the room with the virtual agents during the test phase accounting for effects of social anxiety (C). Total duration spent in the room with the virtual agents during the test phase of Experiment 2 (D), total duration spent with the virtual agents being in the participants' field of view (E) and with accounting for effects of social anxiety (F). Colored ribbons denote standard errors of the mean. Correlations between social anxiety and duration scores are reported separately for the different conditions as Pearson's correlation coefficients.

Table S8

Results of the LMM with the Fixed Effects Condition and Level of Social Anxiety (SPAI) on the Number of Clicks on the Virtual Agents in Experiment 1

Factor	*F*	*df*	*p*	η_p^2	η_p^2 95% CI [LL, UL]
**Experiment 1**					
Condition	11.70	1, 46	**.001**	**.20**	**[.06, .50]**
SPAI	0.17	1, 46	.679	.00	[.00, .01]
Condition × SPAI	0.33	1, 46	.568	.01	[.00, .03]

Note. SPAI = Score of the social phobia and anxiety inventory.

Figure S10

Effect of Social Anxiety and Behavior of the Virtual Agent on Additional Interaction Attempts

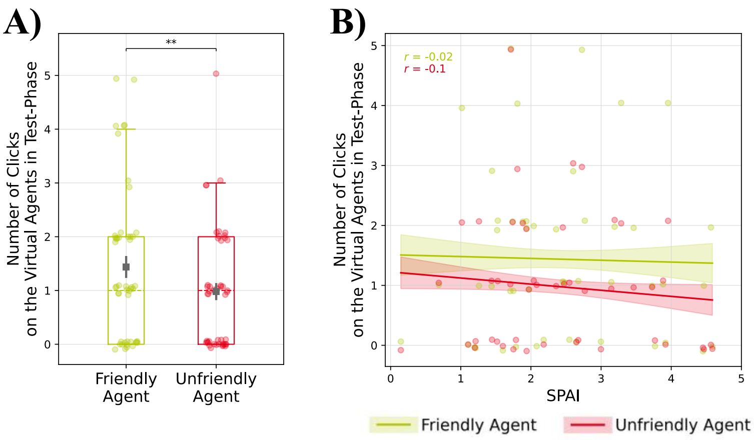

Note. Number of clicks on the virtual agents in Experiment 1 (A) and with accounting for effects of social anxiety (B). Brackets denote significant differences between the conditions.

Physiology

Besides the behavioral analyses, we also examined autonomic responses during the test phase as compared to the habituation phase in the first experiment. Participants showed significantly higher heart rates during the habituation phase compared to the test phase, $F(1, 112.13) = 14.32$, $p < .001$, $\eta_p^2 = .11$ (see Figure S12A). The significant main effect of the condition (see Table S10) should not be interpreted as it combines the habituation and the test phase and a post-hoc *t*-test investigating differences in the heart rates during the test phase revealed no significant effect of condition, $t(111.68) = 0.99$, $p = .324$. For the second experiment, the effect of phase on participants' heart rate failed to reach statistical significance, $F(1, 41) = 3.32$, $p = .076$, $\eta_p^2 = .06$ (Figure S12D). All other main and interaction effects were not statistically significant (see Table S10).

Table S9

Results of the LMM with the Fixed Effects Phase, Condition and Level of Social Anxiety (SPAI) on the Heart Rate

Factor	*F*	*df*	*p*	η_p^2	η_p^2 95% CI [LL, UL]
			**Experiment 1**		
Phase	14.32	1, 112.13	**< .001**	**.11**	**[.03, .33]**
Condition	5.62	1, 111.37	**.019**	**.05**	**[.01, .16]**
SPAI	1.06	1, 41.10	.310	.02	[.01, .09]
Phase × Condition	0.95	1, 112.01	.332	.01	[.00, .03]
Phase × SPAI	0.00	1, 111.28	.953	.00	[.00, .00]
Condition × SPAI	0.02	1, 111.24	.875	.00	[.00, .00]
Phase × Condition × SPAI	0.01	1, 114.05	.904	.00	[.00, .00]
			**Experiment 2**		
Phase	3.32	1, 41	.076	.07	[.02, .24]
Condition[a]	1.95	2, 78.23	.150	.02	[.01, .07]
SPAI[a]	0.57	1, 40.32	.455	.01	[.00, .05]
Condition × SPAI[a]	1.62	2, 78.43	.204	.02	[.01, .06]

Note. SPAI = Score of the social phobia and anxiety inventory.
[a] only considering the test phase.

Regarding skin conductance, there was also a significant main effect of phase in the first experiment, $F(1, 110.0) = 30.12$, $p < .001$, $\eta_p^2 = .21$, with higher skin conductance levels in the test phase as compared to the habituation phase (see Figure S12B). As with the heart rates, the significant main effect of the condition (see Table S11) should not be interpreted as it combines the habituation and the test phase and a post-hoc *t*-test investigating differences in the skin conductance level during the test phase revealed no significant effect of condition,

$t(110.09) = 0.99$, $p = .326$. Additionally, we found a significant interaction between phase and social anxiety, $F(1, 109.92) = 4.19$, $p = .043$, $\eta_p^2 = .04$. Participants with higher trait social anxiety scores had slightly higher skin conductance levels during the test phase ($r_{habituation} = .07$; $r_{test} = .18$). In the second experiment, there was a significant main effect of phase, $F(1, 40) = 21.86$, $p < .001$, $\eta_p^2 = .32$, with higher skin conductance levels in the test compared to the habituation phase (see Figure S12E). We also found a significant main effect of condition in the test phase, $F(2, 76.09) = 3.49$, $p = .035$, $\eta_p^2 = .08$. However, post-hoc *t*-tests only revealed a significant difference between skin conductance measured when being in the same room as the friendly agent compared to when being in a room alone, $t(76.08) = -2.59$, $p = .034$, but no difference between skin conductance measured when being in the same room as the friendly agent compared to when being in the same room as the unfriendly agent, $t(76.12) = 1.75$, $p = .168$. There were no other statistically significant main or interaction effects (see Table S11).

Table S10

Results of the LMM with the Fixed Effects Phase, Condition and Level of Social Anxiety (SPAI) on the Skin Conductance Level

Factor	*F*	*df*	*p*	η_p^2	η_p^2 95% CI [LL, UL]
			**Experiment 1**		
Phase	30.12	1, 110.00	**< .001**	**.21**	**[.06, .51]**
Condition	4.01	1, 109.98	**.048**	**.03**	**[.01, .12]**
SPAI	0.77	1, 39.02	.385	.02	[.00, .07]
Phase × Condition	0.36	1, 110.28	.547	.00	[.00, .01]
Phase × SPAI	4.19	1, 109.92	**.043**	**.04**	**[.01, .13]**
Condition × SPAI	0.35	1, 109.91	.557	.00	[.00, .01]
Phase × Condition × SPAI	0.10	1, 110.98	.750	.00	[.00, .00]
			**Experiment 2**		
Phase	21.86	1, 40	**< .001**	**.34**	**[.11, .68]**
Condition[a]	3.49	2, 76.09	**.035**	**.04**	**[.01, .12]**
SPAI[a]	0.22	1, 39.12	.642	.01	[.00, .02]
Condition × SPAI[a]	0.26	2, 76.16	.772	.00	[.0,0 .01]

Note. SPAI = Score of the social phobia and anxiety inventory.
[a] only considering the test phase.

For pupil diameter, we only found a significant main effect of phase in the second experiment, $F(1, 45) = 25.89$, $p < .001$, $\eta_p^2 = .33$, with participants having wider pupils in the test phase (see Figure S12F). We also found no significant effects on the average pupil diameter in the different phases (see Figure S12C and Table S12).

Table S11

Results of the LMM with the Fixed Effects Phase, Condition and Level of Social Anxiety (SPAI) on the Pupil Diameter

Factor	*F*	*df*	*p*	η_p^2	η_p^2 95% CI [LL, UL]
		**Experiment 1**			
Phase	2.94	1, 127.46	.089	.02	[.01, .08]
Condition	0.89	1, 127.12	.347	.01	[.00, .03]
SPAI	1.30	1, 45.08	.261	.03	[.01, .10]
Phase × Condition	1.02	1, 127.44	.315	.01	[.00, .02]
Phase × SPAI	0.12	1, 127.08	.734	.00	[.00, .00]
Condition × SPAI	0.12	1, 127.04	.727	.00	[.00, .00]
Phase × Condition × SPAI	0.82	1, 128.23	.367	.01	[.00, .02]
		**Experiment 2**			
Phase	25.89	1, 45	**< .001**	**.36**	**[.12, .69]**
Condition[a]	1.87	2, 82.93	.160	.02	[.01, .06]
SPAI[a]	0.36	1, 44.26	.553	.01	[.00, .03]
Condition × SPAI[a]	1.17	2, 82.87	.315	.01	[.00, .04]

Note. SPAI = Score of the social phobia and anxiety inventory.
[a] only considering the test phase.

To summarize, we did not find any long-term adaptations on the physiological level. Thus, participants did not show differential autonomic responses to the virtual agents in the test phase that could solely be attributed to the experimental conditions. We measured slightly lower heart rates and higher skin conductance in the test phase as compared to the initial habituation phase in both experiments, however, we assume that these effects are mainly induced by motion and physical exertion.

Figure S11

Effect of the Behavior of the Virtual Agent on Physiological Responses in the Test Phase

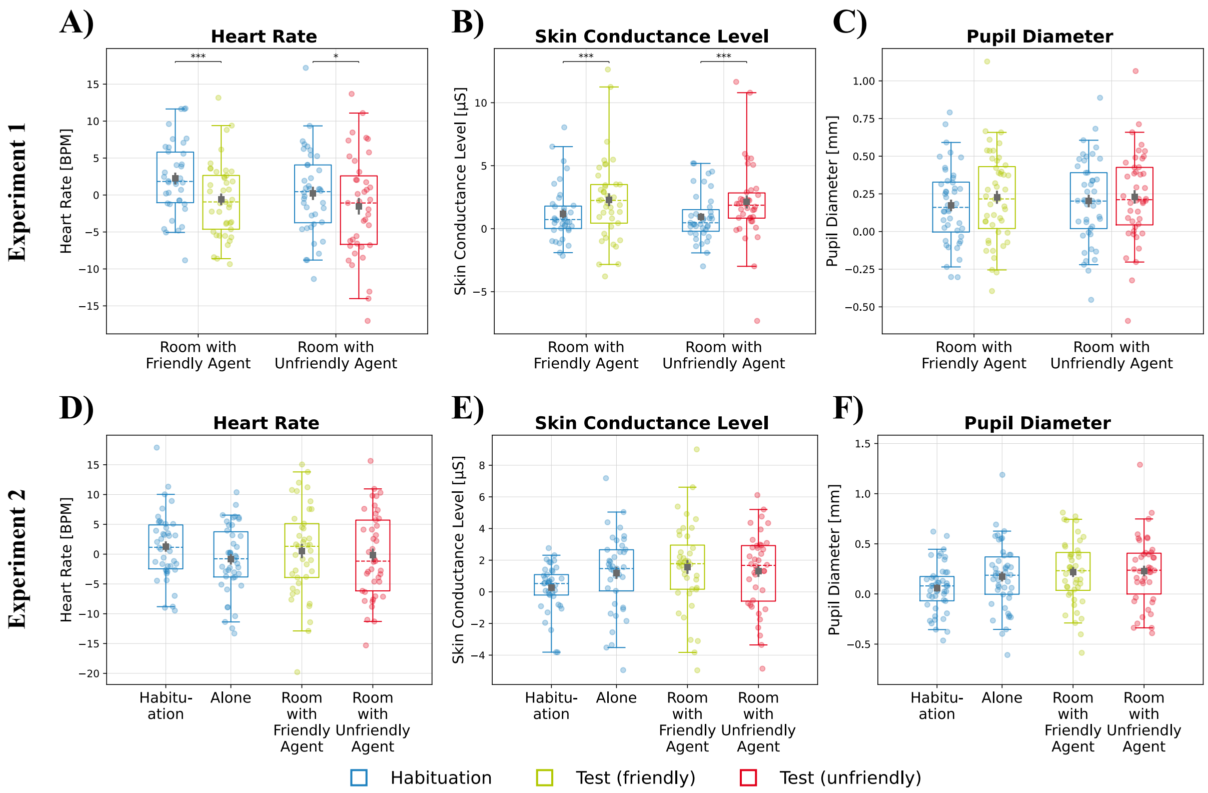

Note. Effects of the previous interactions on heart rate (A), skin conductance level (B) and pupil diameter (C) when being in the same room with virtual agents. Heart Rate: $N = 42$, Skin Conductance Response: $N = 41$, Pupil Diameter: $N = 48$. Brackets denote significant differences between the conditions.

References

Fydrich, T., Scheurich, A., & Kasten, E. (1995). Fragebogen zur sozialen Angst; deutsche Bearbeitung des Social Phobia and Anxiety Inventory (SPAI) von Turner und Beidel. *Psychologisches Institut Der Universität Heidelberg*.

Kennedy, R. S., Lane, N. E., Berbaum, K. S., & Lilienthal, M. G. (1993). Simulator Sickness Questionnaire: An enhanced method for quantifying simulator sickness. *The International Journal of Aviation Psychology*, *3*(3), 203–220. https://doi.org/10.1207/s15327108ijap0303_3

Makransky, G., Lilleholt, L., & Aaby, A. (2017). Development and validation of the Multimodal Presence Scale for virtual reality environments: A confirmatory factor analysis and item response theory approach. *Computers in Human Behavior*, *72*, 276–285. https://doi.org/10.1016/j.chb.2017.02.066

Melo, M., Gonçalves, G., Vasconcelos-Raposo, j., & Bessa, M. (2023). How much presence is enough? Qualitative scales for interpreting the Igroup Presence Questionnaire score. *IEEE Access*, *11*, 24675–24685. https://doi.org/10.1109/ACCESS.2023.3254892

Peters, L. (2000). Discriminant validity of the Social Phobia and Anxiety Inventory (SPAI), the Social Phobia Scale (SPS) and the Social Interaction Anxiety Scale (SIAS). *Behaviour Research and Therapy*, *38*(9), 943–950. https://doi.org/10.1016/S0005-7967(99)00131-X

Sanchez-Vives, M. V., & Slater, M. (2005). From presence to consciousness through virtual reality. *Nature Reviews Neuroscience*, *6*(4), 332–339. https://doi.org/10.1038/nrn1651

Schubert, T., Friedmann, F., & Regenbrecht, H. (2001). The experience of presence: Factor analytic insights. *Presence: Teleoperators and Virtual Environments*, *10*(3), 266–281. https://doi.org/10.1162/105474601300343603

Sheridan, T. B. (1992). Musings on telepresence and virtual presence. *Presence: Teleoperators and Virtual Environments*, *1*(1), 120–126.

Stangier, U., Heidenreich, T., Berardi, A., Golbs, U., & Hoyer, J. (1999). Die Erfassung sozialer Phobie durch die Social Interaction Anxiety Scale (SIAS) und die Social Phobia Scale (SPS). *Zeitschrift Für Klinische Psychologie Und Psychotherapie*, *28*(1), 28–36. https://doi.org/10.1026//0084-5345.28.1.28

Vasser, M., & Aru, J. (2020). Guidelines for immersive virtual reality in psychological research. *Cyberpsychology*, *36*, 71–76. https://doi.org/10.1016/j.copsyc.2020.04.010

Volkmann, T., Wessel, D., Jochems, N., & Franke, T. (2018). *German translation of the Multimodal Presence Scale*. Gesellschaft für Informatik e.V. https://doi.org/10.18420/muc2018-mci-0428

von Glischinski, M., Willutzki, U., Stangier, U., Hiller, W., Hoyer, J., Leibing, E., Leichsenring, F., & Hirschfeld, G. (2018). Optimal Cut Points for Remission and Response for the German Version of the Social Phobia Anxiety Inventory (SPAI). *Zeitschrift Für Psychosomatische Medizin Und Psychotherapie*, *64*(2), 158–171. https://doi.org/10.13109/zptm.2018.64.2.158
